# Synthesis and supramolecular properties of all-*cis*-2,4,6-trifluorocyclohexane-1,3,5-triol†

**DOI:** 10.1039/d3cc05510h

**Published:** 2023-12-11

**Authors:** Shyamkumar V. Haridas, Max von Delius

**Affiliations:** a Institute of Organic Chemistry, Ulm University Albert-Einstein-Allee 11 Ulm 89081 Germany max.vondelius@uni-ulm.de

## Abstract

We report the synthesis of all-*cis* fluorinated cyclohexanes bearing three hydroxy, ether or ester functionalities in the non-fluorinated positions. These tripodal molecules have a high dipole moment of up to 6.3 debye and were successfully used to bind anions and form gels.

## Abstract 2

We report the synthesis of a tripodal, facially polarized cyclohexane building block that is easy to functionalize.

## Graphical abstract

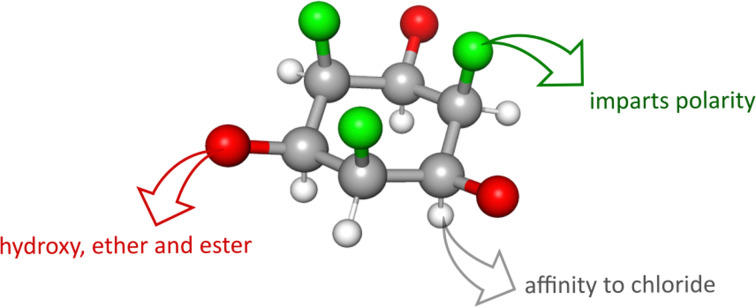

Tripodal molecules are valuable building blocks in supramolecular chemistry. Structural motifs with three-fold symmetry such as amines,^1,2^ orthoesters,^3–5^ 1,3,5 substituted benzenes^6,7^ and cyclohexanes^8^ are therefore used for the synthesis of a variety of cryptands, cages or supramolecular polymers. For the synthesis of receptors and cages it is advantageous when the tripodal molecule provides both a suitable geometry and additional binding affinity with the guest. The orthoester cryptands developed in our lab are ideal examples, because the orthoester bridgehead not only provides the macrobicyclic architecture but also contributes to the binding of cationic guests.

With this in mind, we thought of developing a tripodal architecture based on all-*cis*-1,2,3,4,5,6-hexafluorocyclohexane (all-*cis* C_6_H_6_F_6_).^9,10^ All-*cis* C_6_H_6_F_6_ with its positive and negatively polarized regions and world-record (aliphatic) dipole moment (6.2 debye) holds potential for applications in supramolecular chemistry,^11^ material chemistry^12–15^ and medicinal chemistry^16,17^ (Fig. 1). In 2015 David O’Hagan and co-workers reported the first synthesis of all-*cis* C_6_H_6_F_6_.^9^ Following Glorius’ one-step synthesis of all-*cis* C_6_H_6_F_6_ and its derivatives,^18^ we have studied the anion affinity of this compound class in solution and the solid state^19^ and we have explored the living supramolecular polymerization of fluorinated cyclohexanes.^20,21^

**Fig. 1:**
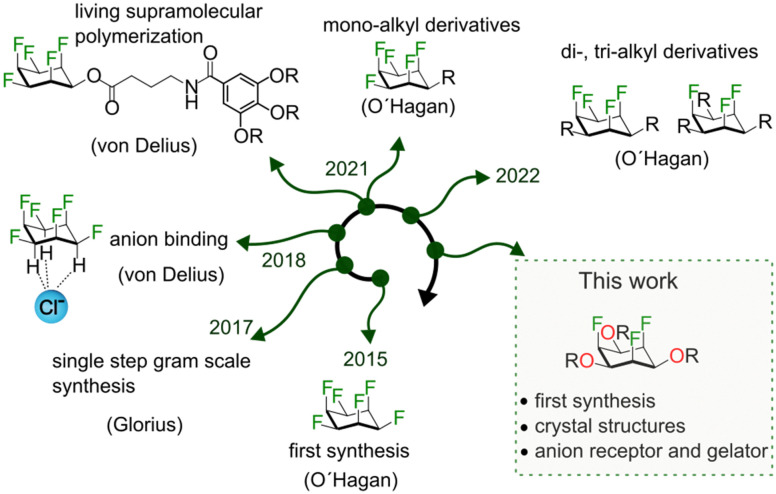
Timeline: synthesis and properties of all-*cis* fluorinated cyclohexanes.

The O’Hagan lab synthesized mono-, di-, and tri-alkyl derivatives of all-*cis* hexafluorocyclohexane.^22–24^ From the solid state structures and thorough physical organic studies the authors demonstrated that it is the three axial fluorine atoms, which are predominantly responsible for the high dipole moment and the solid state packing. With these interesting properties in mind, we wondered whether symmetric tri-oxo derivatives of all-*cis* hexafluorocyclohexanes could be synthesized. In such a molecule, the triaxial fluorine conformation would provide a large dipole moment, while three oxygens on the same face would further increase the dipole moment and represent an opportunity to attach up to three side arms forming a new tripodal motif. Herein, we report the first synthesis of all*-cis*-2,4,6-trifluorocyclohexane-1,3,5-triol and demonstrate the application of this molecule as a gelator and anion receptor.

The trifluorophloroglucinol precursor (1) was synthesized by C–H activation of 1,3,5-trifluorobenzene using bis(pinacolato)diboron (B_2_Pin_2_), (1,5-cyclooctadiene)(methoxy)iridium(i) dimer as catalyst and 4,4′-di-*tert*-butyl-2,2′-dipyridyl (dtbPy) as ligand according to a reported procedure.^25^ The obtained triborylated compound was then oxidized using oxone to obtain the trifluorophloroglucinol 1.^26^ The hydroxy groups were protected by treatment with methyl iodide. The crucial face-selective hydrogenation step was carried out in a steel reactor using the rhodium–cyclic (alkyl)(amino)carbene (RhCAAC) catalyst^18^ and hydrogen (60 bar) at 50 °C for 14 days to give 2 in 20–40% yield. In a typical reaction, we obtained *ca.* 30% yield of the product and recovered *ca.* 30% of the starting material. Additionally, we observed the formation of some side products (possibly partially hydrogenated/defluorinated compounds^18^), but an exact identification of these minor side products is not straightforward. Single crystal XRay diffraction (SCXRD) of trimethoxy ether 2 revealed an axial conformation of the three fluorine atoms and conversely the equatorial conformation for the methoxy groups with parallel stacks of cyclohexanes in the *P*6_3_ space group (Fig. 2). The axial fluorine atoms in one molecule point towards the center of two axial hydrogen atoms (Fig. 2b) in a neighbouring molecule with a CF–HC distance of 2.46 Å, which is below the sum of vdW radii of the two atoms (2.67 Å) and in accordance with a related structure.^22^ The non-centrosymmetric nature of the space group points toward potential applications of this compound class in ferroelectric and piezoelectric materials.

**Fig. 2:**
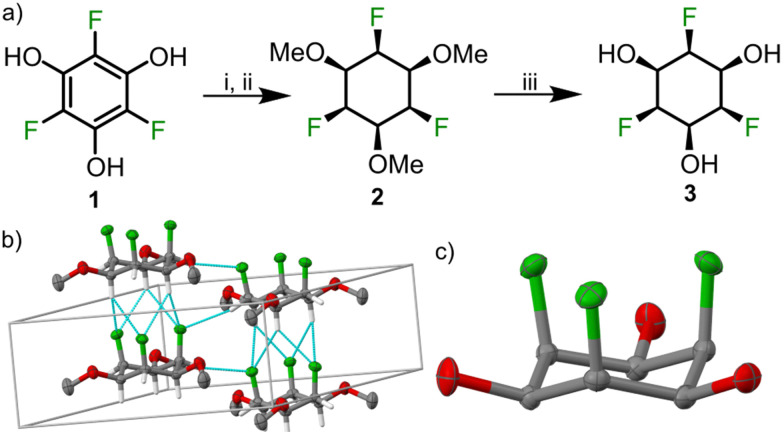
(a) Synthesis of triol 3: (i) CH_3_I, K_2_CO_3_, DMF, 80 °C, 12 h, 78%, (ii) RhCAAC catalyst, H_2_ (60 bar), hexane, 50 °C, 14 d, 20–40%, (iii) AlCl_3_, *n*-BuSH, CH_2_Cl_2_, rt, 12 h, 48%. (b) Solid state structure and crystal packing of 2 (space group *P*6_3_, crystallized by slow evaporation from acetone solution); F–H contacts between cyclohexanes are shown as cyan lines; methyl H atoms are omitted for clarity. (c) Solid state structure of 3 (recrystallized from acetone, space group *Pnma*). Colour code: oxygen: red; fluorine: green; carbon: grey. Ellipsoids are shown at 50% probability.

To complete the synthesis of the desired all*-cis*-2,4,6-trifluorocyclohexane-1,3,5-triol 3, the methoxy ethers were cleaved using aluminium trichloride and *n*-butanethiol. SCXRD of 3 revealed a solid-state structure in which stacks of cyclohexanes interact *via* axial fluorine atoms, axial hydrogen atoms and equatorial oxygen atoms. A molecule of water was present in the asymmetric unit cell and involved in hydrogen bonding with the hydroxyl group of 3. Due to the centrosymmetric nature of the space group *Pnma*, stacks of the fluorinated cyclohenaxes are arranged in opposite direction, which is contrast to the packing observed for 2, where all stacks are arranged in the same direction (see Fig. S1 and S2, ESI†).

Having successfully synthesized compounds 2 and 3 we were interested in their dipole moments and “ring-flip” equilibria, which is why we performed high-level DFT calculations (B3LYP-D3/def2-TZVP and PBE0-D3/def2-TZVP) and VT-NMR experiments (details are provided in the ESI† file, including link to the raw DFT data). As summarized in Fig. 3, we found that 2 in the F-axial conformation has a dipole moment of 6.3 debye, which is very similar to the parent molecule all-*cis* hexafluorocyclohexane.^9^ The dipole moment of 2 is significantly lower in the F-equatorial conformation, which is expected from the difference of electronegativity between fluorine and oxygen. For compound 3 we observed the opposite trend, however, *i.e.* higher dipole moment for the F-equatorial conformer and we propose that this is due to the presence of relatively strong intramolecular hydrogen bonds between neighbouring OH and F groups, as described in reports on related systems.^27,28^ The hypothesis is further corroborated by the calculated free energies of axial-*vs*.-equatorial conformers in the gas-phase (F-axial 3 is an outlier that exhibits six F–HO hydrogen bonds, see Fig. S3, ESI†). Since the calculated gas-phase energy difference between the conformers of 2 was only 0.8 kcal mol^−1^, we attempted to observe the ring flip isomers by low temperature NMR spectroscopy. However, even at a temperature of −80 °C in acetone, when coalescence should have occurred,^9,10^ we could only observe one signal in the ^19^F NMR spectrum (Fig. S9, ESI†). This finding indicates that the DFT calculations that take solvent into account, correctly predict the strong thermodynamic preference for the F-axial isomers.

**Fig. 3:**
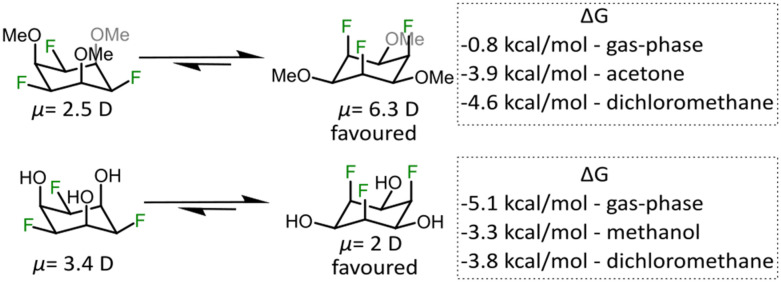
Calculated dipole moments for “ring flip” conformers of 2 and 3 and the free energy difference in the gas-phase and two implicit solvents. Level of theory: DFT, PBE0-D3/def2-TZVP (for dipole moment and single point energy calculations), in line with previous computational work on related molecules.^23,24^

Having understood key structural aspects of the title compounds, we decided to explore supramolecular applications of compound 3 and its simple derivatives. We thought that simple amphiphiles could be made by alkylating one of the hydroxyl groups with a non-polar alkyl chain. Amphiphiles^29^ are used as gelators since they are known to form cylindrical micelles, and further bundling will lead to the formation of supramolecular gels.^30–33^ We synthesized monoalkyl derivative 4 by reacting 3 with the pentafluorophenol (PFP) active ester of dodecanoic acid (Fig. 4a). As expected, 4 formed a gel in toluene (0.75 wt%) and scanning electron microscopic (SEM) revealed a fibrous morphology (Fig. 4e). The role of hydroxyl groups and the fluorines in self assembly was confirmed by the SCXRD data of a model compound 5 (shorter alkyl chain (Fig. 4b and c)). We propose that such networks could give rise to interesting ferroelectric^34^ behaviour.

**Fig. 4:**
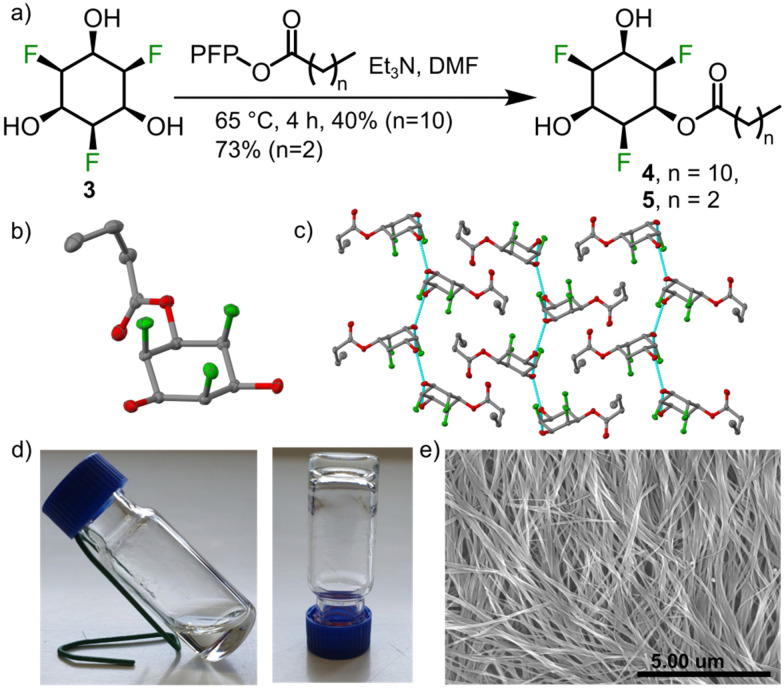
(a) Synthesis of esters 4 and 5. (b) Solid state structure of 5 (single crystal obtained from methanol layered with *n*-hexane, space group *p*2_1_/*n*; colour code and ellipsoids as in Fig. 2). (c) Hydrogen bonds (cyan lines) observed in the solid state packing of 5. (d) 4 forms a gel at 0.75 wt% in toluene. (e) SEM image of the toluene gel of 4 showing fibrous morphology.

We also decided to synthesize simple anion receptors^35–42^ from triol 3, in order to make use of the known anion affinity of all-*cis* fluorinated cyclohexanes (binding constants 400 M^−1^ for the parent compound and 170 M^−1^ for a mono-derivative determined in acetone^19^). For the tris-methoxyether 2 we determined a chloride binding constant of 25 M^−1^ in acetone (Fig. S8 and Table S5, ESI†) which shows that alkoxy-substitution leads to decreased binding affinity. To investigate whether higher binding constants can be obtained in tripodal architectures, we synthesized new receptors 8 and 9 (Fig. 5). We used CuAAC “click” chemistry, because the resulting triazole motifs can provide additional C–X (X = H or I) hydrogen or halogen bonding^43,44^ to the anion. The hydroxyl groups of 3 were functionalized using propargyl bromide and sodium hydride in DMF to obtain the trialkyne 6 (Fig. 5a). 6 was treated with phenyliodine(iii)diacetate (PIDA) and tetrabutylammonium iodide (TBAI) in acetonitrile furnishing the triiodo alkyne compound 7. Tris-triazole receptor 8 and tris-iodotriazole receptor 9 were obtained by reacting benzyl azide with 6, 7 respectively. NMR host–guest titrations (ESI†) were carried out with both hosts (8, 9) in CH_2_Cl_2_ using tetrabutylammonium chloride (TBACl) as chloride ion source. Both receptors showed anion affinity higher than 2 (in CH_2_Cl_2_), which is most likely due to the presence of additional C–X bonds to the anion. Receptor 8 binds to Cl^−^ with a *K*_a_ of 29 M^−1^ while receptor 9 binds with a *K*_a_ of 142 M^−1^ (Fig. 5c). We propose that the higher *K*_a_ observed for 9 (compared to 8) is due to halogen bonding and the larger size of the three iodine atoms, which probably helps the XB donor to “reach” the chloride ions in the tripodal binding cavity. Overall, the relatively low association constants can be explained by the open and relatively flexible geometry of the receptors.

**Fig. 5:**
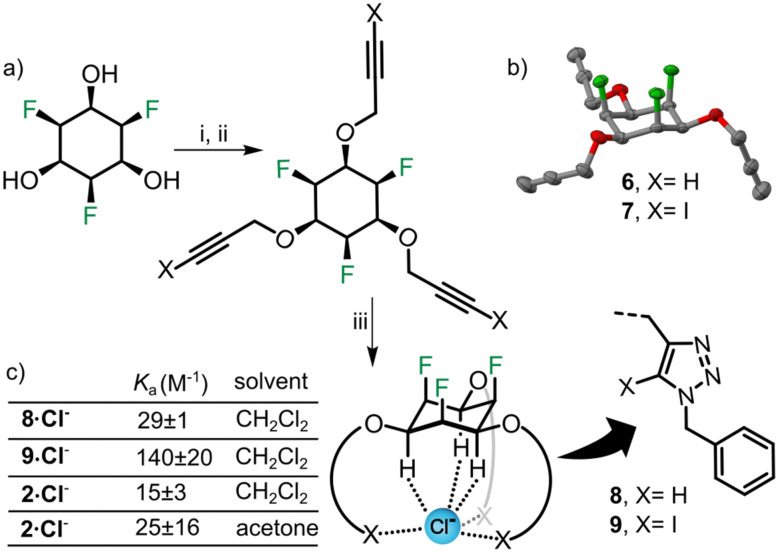
(a) (i) for the synthesis of 6-NaH, propargyl bromide, DMF, 0 °C to rt, overnight, 62% (ii) for the synthesis of 7-alkyne 6, PIDA, TBAI, ACN, rt 2 days, 91%, (iii) (Cu(CH_3_CN)_4_)PF_6_, TBTA, benzyl azide, THF, rt, overnight, 8 in 63% and 9 in 55%. (b) Crystal structure of 6; colour code and ellipsoids as in Fig. 2 and 4. (c) Association constants of 8, 9 and 2 for the chloride ion (as TBACl salt) in different solvents (standard error based on triplicate experiments).

In conclusion, we synthesized all*-cis*-2,4,6-trifluorocyclohexane-1,3,5-triol and demonstrate its potential applications as a tripodal molecule. DFT calculations showed the dipole moment is especially large, when three axial fluorine atoms are complemented by three equatorial OR groups (R ≠ H) on the same face. We were able to make use of this dipole moment in proof-of-principle studies on a simple gelator and a tripodal receptor for the chloride ion (whose binding affinity was enhanced by hydrogen/halogen bonding with triazole/iodotriazole side arms). These results show the potential of this tripodal and highly polar platform in a variety of supramolecular applications. Future work will focus on the synthesis of rigid dynamic covalent^45^ as well as metalla cages^46^ and the exploration of ferroelectric^34^ behaviour.

This work was supported by the European Union (ERCstg 802428 “SUPRANET”). The authors acknowledge the infrastructure provided by the state of Baden-Württemberg through bwHPC and DFG through grant no. INST 40/575-1 FUGG (JUSTUS 2 cluster) and Mohammed Siddhique for his valuable suggestions in DFT calculations.

## Conflicts of interest

There are no conflicts to declare.

## Supplementary Material

CC-060-D3CC05510H-s001

CC-060-D3CC05510H-s002
