# Supplementary material for: Synthesis and supramolecular properties of all-cis-2,4,6-trifluorocyclohexane-1,3,5-triol
Source: Chem Commun (Camb). 2023 Dec 11;60(5):606–9. doi: 10.1039/d3cc05510h (PMC10783651; doi:10.1039/d3cc05510h)
Supplement: CC-060-D3CC05510H-s001 [file CC-060-D3CC05510H-s001.pdf]

Electronic Supplementary Material (ESI) for ChemComm.
This journal is © The Royal Society of Chemistry 2023

Electronic supplementary information

Synthesis and supramolecular properties of all-*cis*-2,4,6-trifluorocyclohexane-1,3,5-triol

Shyamkumar V. Haridas, Max von Delius*

Institute of Organic Chemistry, Ulm University, Albert-Einstein-Allee 11, 89081 Ulm, Germany

E-Mail: max.vondelius@uni-ulm.de

Table of Contents

1. General methods 3
2. Synthesis of $C_6H_6F_3(OR)_3$ derivatives 4
3. Supplementary figures 16
4. DFT calculations 17
5. NMR host-guest titrations 23
6. VT-NMR experiment 32
7. Characterization data 33
8. Crystallographic data 47
9. References 52

1. General methods

All commercially available reagents and solvents were purchased from Merck- Sigma Aldrich, ABCR, TCI Germany and were used without further purification. Hydrogenation reactions were carried out in a 150 mL Roth High pressure steel reactor. NMR spectra were recorded on a BrukerAvance 600 (^{1}H: 600 MHz; ^{13}C: 151 MHz) and BrukerAvance 400 (^{1}H: 400 MHz; ^{13}C: 126 MHz, ^{19}F: 376 MHz) spectrometers at 298 K and referenced to the residual solvent peak (^{1}H: chloroform- *d*, 7.26 ppm; acetone-d_6, 2.05 ppm; dichloromethane-d_2, 5.32 ppm; methanol-d_4, 3.31 ppm; ^{13}C: chloroform-*d*, 77.6 ppm; acetone-d_6, 206.68 ppm; methanol-d_4, 49 ppm; dichloromethane-d_2, 53.84 ppm). Coupling constants (J) are denoted in Hz and chemical shifts (δ) in ppm. Multiplicities are denoted as follows: s = singlet, d = doublet, t = triplet, m = multiplet, br = broad. High-resolution mass spectrometry (HRMS) was performed using a Fourier Transform Ion Cyclotron Resonance (FT-ICR) mass spectrometer solariX (Bruker Daltonik GmbH, Bremen, Germany) equipped with a 7.0 T superconducting magnet and interfaced to an Apollo II Dual ESI/MALDI source (using *trans*-2[3-(4-*tert*-butylphenyl)-2methyl-2-propenylidene]malononitrile (DCTB) as matrix for MALDI), which can be switched from ESI/APCI to MALDI operation almost instantaneously. Analytical thin layer chromatography was used to monitor reactions on pre-coated aluminium plates and visualized by dipping in $KMnO_4$ stain followed by heating.

2. Synthesis of $C_6H_6F_3(OR)_3$ derivatives

Overview of synthesis

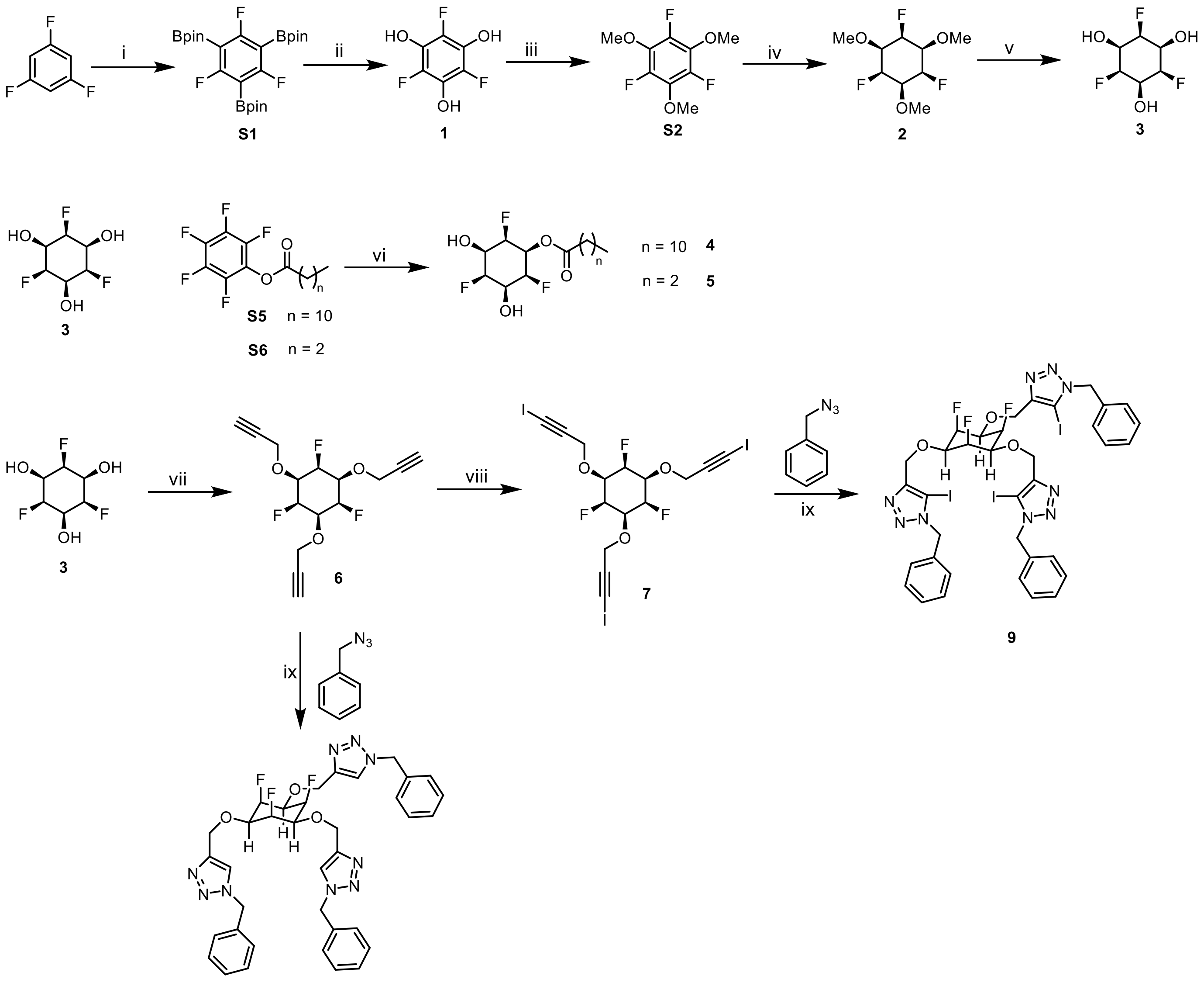

Scheme 1 i) Bis(pinacolato)diboron (B_2Pin_2), (1,5-cyclooctadiene)(methoxy)iridium(I) dimer, 4,4´-di-tert-butyl-2,2´-dipyridyl (dtbPy), THF, reflux, overnight. ii) Oxone, acetone, water, 72 % combined yield for step i and ii. iii) Methyl iodide, K_2CO_3, DMF, 80 °C, overnight, 78%. iv) RhCAAC catalyst, molecular sieves, hexane, H_2 (60 bar), 50 °C, 14 days, 20-40 %. v) $AlCl_3$, *n*-BuSH, DCM, rt, overnight, 48%. vi) Et_3N, DMF, 65 °C, 4 h, 40% for **5**, 73% for **4**. vii) NaH, propargyl bromide, 0 °C to rt, overnight, 62%. viii) Phenyliodine(III)diacetate (PIDA), tetrabutylammonium iodide (TBAI), acetonitrile, rt, 2 days, 91%. ix) Tetrakis acetonitrile copper hexafluorophosphate, tris(benzyltriazolylmethyl)amine, THF, rt, overnight, 63% for **8** and 55% for **9**.

2,4,6-trifluorobenzene-1,3,5-triol (1)

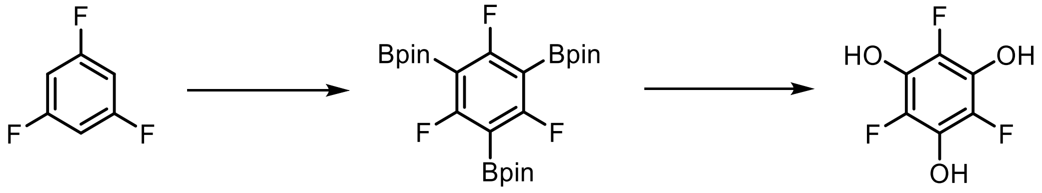

S1 **1**

The triborylated compound **S1** was synthesized according to a reported procedure[1]. Oxidation of **S1** was carried out using Oxone according to a reported procedure[2].

A Schlenk flask was charged with B_2Pin_2 (12.7 g, 50 mmol, 5 eq), $[IrOMe(COD)]_2$ (200 mg, 0.3 mmol, 3 mol %), dtBPy (160 mg, 0.6 mmol, 6 mol%) and dried under high vacuum for 2 h. Then THF (50 mL) was added and 1,3,5-trifluorobenzene was also added. The reaction was refluxed overnight under the atmosphere of nitrogen. The solvents were then evaporated and, after cooling to RT, hexane (150 mL) was added to precipitate the product. After washing with hexane, the solid was dried under reduced pressure.

The crude product (**S1**) was dissolved in 90 mL acetone and Oxone (11.2g) dissolved in 80 mL of water was added to the acetone solution dropwise with vigorous stirring, and stirring continued for another fifteen minutes after complete addition of Oxone solution. Ethyl acetate was added and the organic layer was separated. The aqueous layer was washed with ethyl acetate two times. The combined organic layer was washed with brine and dried over $MgSO_4$. The organic solvents were evaporated under reduced pressure. A yellowish, thick oil was obtained. Dichloromethane (80 mL) was added to precipitate the product **1**. Dichloromethane was decanted and the process was repeated for 2-3 times. The solid was dried under reduced pressure to obtain the final compound **1** (72 % yield) as yellow solid. Characterization data matched the reported data[2].

1,3,5-trifluoro-2,4,6-trimethoxybenzene (S2)

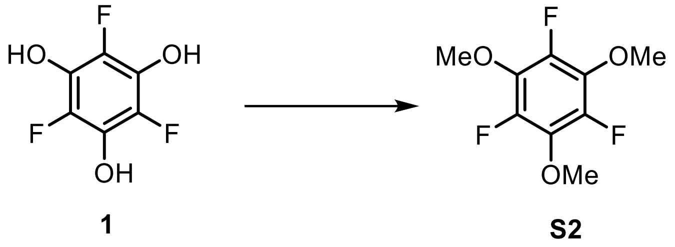

1 (1g, 5.6 mmol, 1 eq) was dissolved in 100 mL of DMF and methyl iodide (10.3 mL, 168 mmol, 30 eq) was added to it. Potassium carbonate (4.6 g, 33.6 mmol, 6 eq) was added portion wise while stirring the solution. The reaction was then stirred at 80 °C overnight. Water was added to the reaction and extracted with ethyl acetate three times. The combined organic layer was then washed with water and dried over $MgSO_4$. The solvents were evaporated under reduced pressure. Column chromatography was performed using 1:19 ethyl acetate / petroleum ether to obtain **S2** as a colourless liquid that solidified upon standing (980 mg, 78%).

^{1}H NMR (400 MHz, 298 K, acetone-d_6) δ 3.94 (t, J = 0.9 Hz, 9H).

^{13}C NMR (126 MHz, 298 K, acetone-d_6) δ 146.69 (d, split due to ^{13}C/^{19}F coupling, J = 243.5 Hz), 135.29, 62.91.

^{19}F NMR (376 MHz, 298 K, acetone-d_6) δ -154.31 (s, 3F).

HR-MS (APCI)- m/z = Cal. For $C_9H_9F_3O_3$: 222.0504, observed: 222.0496 $[M]^+$.

All-*cis*-1,3,5-trifluoro-2,4,6-trimethoxycyclohexane (2)

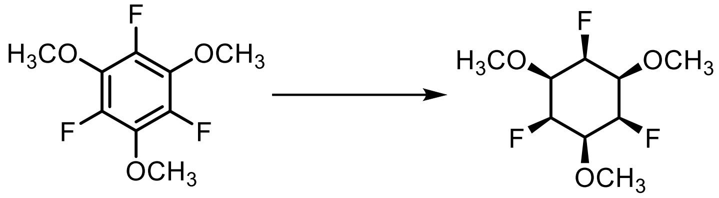

S2 **2**

The catalyst for hydrogenation was synthesized according to the known procedure[3]. The catalyst (109 mg, 5 mol %) and activated molecular sieves (2 g) were added to a cylindrical glass tube. The glass tube was then kept under vacuum for 30 minutes. Dry hexane (40 mL) and **S2** (750 mg, 3.4 mmol) dissolved in hexane was added to the glass tube and placed in a high-pressure steel reactor and 10 cycles of vacuum and nitrogen were applied. The steel reactor was purged with hydrogen three times (20 bar). Finally the steel reactor was pressurized with hydrogen (60 bar) and stirred for 2 weeks at 50 °C. The steel reactor was depressurized and the reaction mixture was filtered and the solvents were evaporated under reduced pressure. Column chromatography was performed using 4:6 acetone / dichloromethane to obtain **2** as a colourless, fluffy solid (20-40% yield).

Rf = 0.36 in 1:4 Acetone, Dichloromethane eluent system.

Single crystals suitable for X-ray diffraction were obtained by slow evaporation of an acetone solution.

Melting point is 238 °C

^{1}H NMR (600 MHz, 298 K, chloroform-*d*) δ 5.29 – 5.08 (m, 3H), 3.58 (s, 9H), 3.18 (m, 3H).

^{13}C NMR (151 MHz, 298 K, chloroform-*d*) δ 85.63 (d, split due to ^{13}C/^{19}F coupling, J = 194.5 Hz), 75.93, 57.22.

^{19}F NMR (376 MHz, 298 K, chloroform-*d*) δ -214.38 (s, 3F).

HR-MS (APCI)- m/z = Cal. For $C_9H_{16}F_3O_3$: 229.1052, observed: 229.1044 $[M+H]^+$.

All-*cis*-2,4,6-trifluorocyclohexane-1,3,5-triol (3)

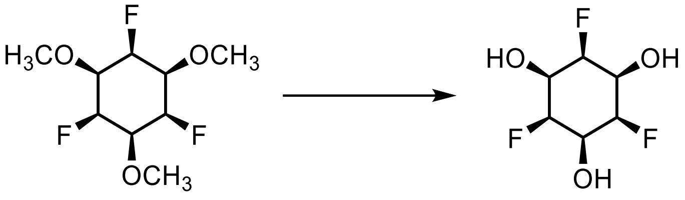

2 **3**

2 (230 mg, 1 mmol, 1 eq) was dissolved in 15 mL dichloromethane. $AlCl_3$ (1.4 g, 10.5 mmol, 10.5 eq) and 1-butane thiol (3.2 mL, 30 mmol, 30 eq) were added to the dichloromethane solution at 0 °C and the reaction was stirred overnight, while bringing the temperature slowly to room temperature. Methanol and water was added dropwise to the reaction at 0 °C to quench the unreacted $AlCl_3$. The solvents were then evaporated and column chromatography (dry loading) was performed using 3:2 acetone / dichloromethane to obtain **3** as a colourless, fluffy solid (90 mg, 48%).

Rf = 0.3 (with tailing) in 3:2 Acetone, Dichloromethane eluent system.

Melting point is 135 °C

Single crystals suitable for X-ray diffraction were obtained by recrystallization from acetone.

^{1}H NMR (600 MHz, 298 K methanol-d_4) δ 4.82 – 4.66 (m, 3H), 3.79 – 3.63 (m, 3H).

^{13}C NMR (151 MHz, 298 K, methanol-d_4) δ 93.24 (d**,** split due to ^{13}C/^{19}F coupling, J = 186 Hz), 68.43.

^{19}F NMR (376 MHz, 298 K, methanol-d_4) δ -217.05 (s, 3F).

ATR-IR solid state: 3350, 1433, 1152, 1070, 948, 880, 775, 558, 482 cm^{-1}

HR-MS (APCI)- m/z = Cal. For $C_6H_{10}F_3O_3$: 187.0582, observed: 187.0575 $[M+H]^+$.

General procedure for the synthesis of active ester S5 and S6

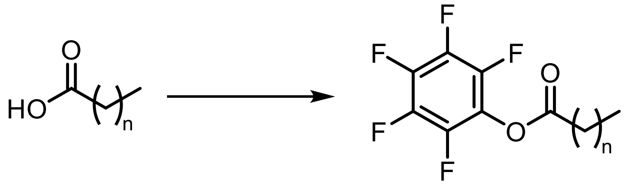

S3, n=10
S4, n=2

S5, n=10
S6, n=2

To a dichloromethane solution of corresponding acid **S3/S4** (1 eq) was added EDC.HCl (1.1 eq) and pentafluorophenol (PFP, 1.1 eq) and 4-dimethylaminopyridine (DMAP 0.1 eq) as catalyst. The reaction was stirred at room temperature over night. The solution was washed with water 4 times and then with brine one time and the organic layer was dried over $MgSO_4$. The organic solvent evaporated under reduced pressure to give the active ester (**S5/S6**) in quantitative yield. The active ester was used without further purification for the next step.

The NMR spectra of these compounds were in good agreement with the reported data[4,5].

General procedure for the synthesis of 4 and 5

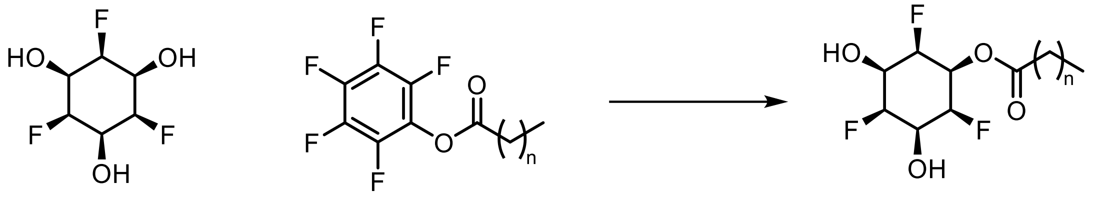

3 **S5, n= 10** **S6, n=2** **4, n= 10** **5, n=2**

To a 10 mL DMF solution of **3** (30 mg, 0.16mmol, 1 eq) was added the active ester (**S5/S6**) (1 eq) and 8 mL of triethylamine. The reaction was refluxed at 65 °C for 4 h. After cooling, water and ethyl acetate were added. The aqueous layer was extracted with ethyl acetate three times and then with dichloromethane 3 times. The organic layers were combined and dried over $MgSO_4$. The solvents were then evaporated under reduced pressure. Column chromatography was performed (see below).

(1s,2R,3R,4r,5S,6S)-2,4,6-trifluoro-3,5-dihydroxycyclohexyl dodecanoate (4): column chromatography using 1:4 acetone / dichloromethane (yield: 40%).

Melting point is 180 °C

^{1}H NMR (600 MHz, 298 K, acetone-d_6) δ 5.10 – 4.78 (m, 4H), 4.69 (d, J = 9.5 Hz, 2H), 4.05 – 3.88 (m, 3H), 2.43 (t, J = 7.4 Hz, 2H), 1.65 (p, J = 7.4 Hz, 2H), 1.40 – 1.23 (m, 16H), 0.88 (t, 3H).

^{13}C NMR (151 MHz, 298 K, acetone-d_6) δ 172.20, 92.3 (d, split due to $^{13}C/^{19}F$ coupling, J = 186.1 Hz), 89.71 (split due to $^{13}C/^{19}F$ coupling, J = 186.1 Hz), 68.51, 67.07, 33.52, 31.73, 24.73, 22.42, 13.44.

^{19}F NMR (376 MHz, 298 K, acetone-d_6) δ -215.10 (d, J = 22.3 Hz, 2F), -216.77 (t, J = 22.3 Hz, 1F).

HR-MS (APCI)- m/z = Cal. For $C_{18}H_{34}F_3O_5$: 387.2358, observed: 387.2350 $[M+H_3O]^+$.

(1s,2R,3R,4r,5S,6S)-2,4,6-trifluoro-3,5-dihydroxycyclohexyl butyrate (5): column chromatography using 1:1 acetone / dichloromethane (yield: 73%)

Melting point is 210 °C

^{1}H NMR (600 MHz, 298 K, acetone-d_6) δ 5.10 – 4.77 (m, 4H), 4.69 (d, J = 9.5 Hz, 2H), 4.06 – 3.87 (m, 3H), 2.41 (t, J = 7.3 Hz, 2H), 2.08-2.02 a CH2 peak merged with acetone solvent peak, 1.66 (h, J = 7.4 Hz, 2H), 0.96 (t, J = 7.4 Hz, 3H).

^{13}C NMR (151 MHz, 298 K, acetone-d_6) δ 172.95, 93.21 (split due to $^{13}C/^{19}F$ coupling, J = 186.1 Hz), 90.61 (split due to $^{13}C/^{19}F$ coupling, J = 193.2 Hz), 69.42, 67.97, 36.25, 19.04, 13.72.

^{19}F NMR (376 MHz, 298 K, acetone-d_6) δ -215.14 (d, *J* = 22.3 Hz, 2F), -216.77 (t, *J* = 22.4 Hz, 1 F).

HR-MS (APCI)- m/z = Cal. For $C_{10}H_{18}F_3O_5$: 275.1106, observed: 275.1098 $[M+H_3O]^+$

Synthesis of all-*cis*-1,3,5-trifluoro-2,4,6-tris(prop-2-yn-1-yloxy)cyclohexane (6)

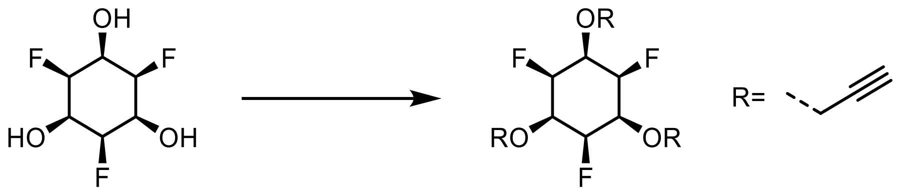

3 **6**

To a 50 mL DMF solution of **3** (100 mg, 0.54 mmol, 1 eq) was added NaH (60% in mineral acid, 78 mg of 3.6 eq) at 0 °C. Propargyl bromide (1.94 mmol, 3.6 eq) dissolved in 10 mL DMF was added dropwise using an addition funnel at 0 °C. The reaction was allowed to warm to room temperature slowly and stirred overnight. The unreacted NaH was quenched carefully by adding ice. Water was added and the mixture was extracted using dichloromethane three times. The organic fractions were then dried over $MgSO_4$ and evaporated. Column chromatography was performed using 95:5 dichloromethane / acetone to furnish **6** as a colourless solid (90 mg, 62%).

Melting point is 175 °C

^{1}H NMR (600 MHz, 298 K, acetone-d_6) δ 5.31 – 5.11 (m, 3H), 4.42 (d, J = 2.4 Hz, 6H), 4.02 – 3.84 (m, 3H), 3.02 (t, J = 2.4 Hz, 3H).

^{13}C NMR (151 MHz, 298 K, acetone-d_6) δ 87.68 (d, split due to ^{13}C/^{19}F coupling, J = 193.1 Hz), 80.23, 76.78, 73.91, 56.78.

^{19}F NMR (376 MHz, 298 K, acetone-d_6) δ -214.58 (3F).

HR-MS (APCI)- m/z = Cal. For $C_{10}H_{18}F_3O_5$: 301.1046, observed: 301.1045 $[M+H]^+$.

All-*cis*-1,3,5-trifluoro-2,4,6-tris((3-iodoprop-2-yn-1-yl)oxy)cyclohexane (7)

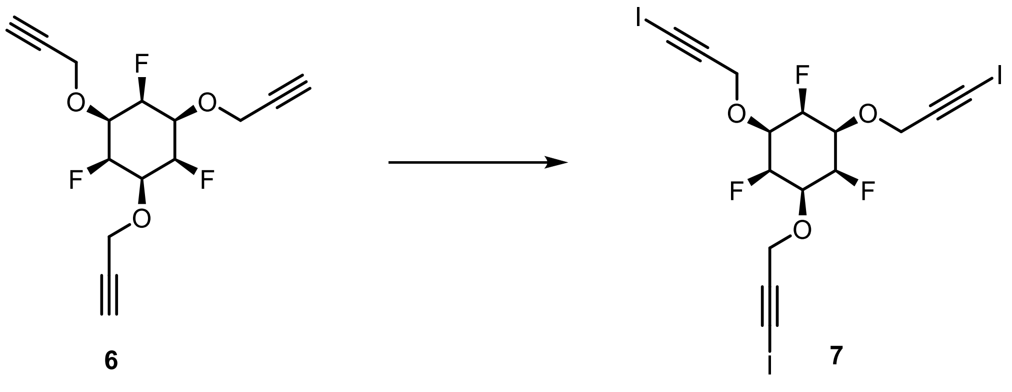

The reaction was performed according to a reported procedure [6].

6 (70 mg, 0.23 mmol, 1 eq) was dissolved in 12 mL acetonitrile and tetrabutyl ammonium iodide (3.6 eq) was added. To that solution diacetoxyiodobenzene (PIDA, 5 eq) was added in portions over fifteen minutes. The reaction was monitored by TLC. After completion dichloromethane was added to the reaction and washed with sodium thiosulphate solution, water and brine. The organic layer was then dried over $MgSO_4$. After evaporation of the organic solvents und reduced pressure, column chromatography was performed using a dichloromethane / acetone gradient (starting from 100 % dichloromethane to 95:5) to give the triiodo alkyne **8** in 91% yield.

Melting point is 190 °C and colour change from white to yellow brown above this temperature.

^{1}H NMR (600 MHz, 298 K, acetone-d_6) δ 5.29 – 5.09 (m, 3H), 4.56 (s, 6H), 4.00 – 3.84 (m, 3H).

^{13}C NMR (151 MHz, 298 K, acetone-d_6) δ 90.58, 87.54 (d, split due to ^{13}C/^{19}F coupling, J = 193.3 Hz), 73.94, 58.26, 8.01.

^{19}F NMR (376 MHz, 298 K, acetone-d_6) δ -214.53.

HR-MS (APCI)- m/z = Cal. For $C_{15}H_{13}F_3O_3I_3$: 678.7951, observed: 678:7958 $[M+H]^+$.

4,4',4''-((((1s,2s,3s,4s,5s,6s)-2,4,6-trifluorocyclohexane-1,3,5-triyl)tris(oxy))tris(methylene))tris(1-benzyl-1H-1,2,3-triazole) (8)

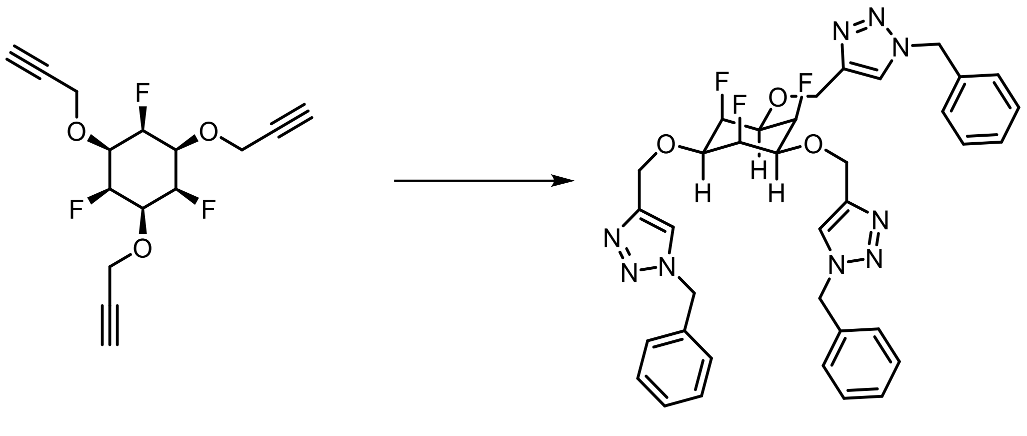

6 8

An oven dried Schlenk flask was charged with tetrakis acetonitrile copper hexafluorophosphate (227 mg, 0.61 mmol, 2.25 eq) and tris(benzyltriazolylmethyl)amine (TBTA) (324 mg, 0.61 mmol, 2.25 eq). The flask was evacuated and filled with nitrogen three times. Dry THF (5 mL, degassed with nitrogen for fifteen minutes) was added to the flask and stirred for fifteen minutes. In another flask **6** (80 mg, 0.27 mmol, 1 eq) and benzyl azide (160 mg, 1.2 mmol, 4.5 eq) was dissolved in 5 mL THF and degassed with nitrogen for ten minutes. This mixture was added to the Schlenk flask dropwise and the reaction was stirred over night at room temperature. Ammonia solution (2 %-volume) was added and extracted with DCM. The combined organic layer was washed with brine and dried over $MgSO_4$. The solvent was evaporated under reduced pressure. Column chromatography was performed using acetone / methanol gradient (starting from 100% acetone to remove TBTA, then 5:1 acetone / methanol) to furnish **10** as a colourless solid (120 mg, 63%).

Melting point is 217 °C

^{1}H NMR (600 MHz, 298 K, dichloromethane-d_2) δ 7.63 (s, 3H), 7.40 – 7.27 (m, 15H), 5.53 (s, 6H), 5.13 – 4.93 (m, 3H), 4.77 (s, 6H), 3.52 – 3.37 (m, 3H).

^{13}C NMR (151 MHz, 298 K, dichloromethane-d_2) δ 144.57, 135.24, 129.47, 129.10, 128.56, 123.75, 87.19 (d, split due to ^{13}C/^{19}F coupling, J = 193 Hz) , 73.34, 62.45, 54.58, 54.22.

^{19}F NMR (376 MHz, 298 K, dichloromethane-d_2) δ -214.86.

HR-MS (MALDI)- m/z = Cal. For $C_{36}H_{37}F_3O_3N_9$: 700.2971, observed: 700.2962[M+H]$^+$.

4,4',4''-((((1s,2s,3s,4s,5s,6s)-2,4,6-trifluorocyclohexane-1,3,5-triyl)tris(oxy))tris(methylene))tris(1-benzyl-5-iodo-1H-1,2,3-triazole) (9)

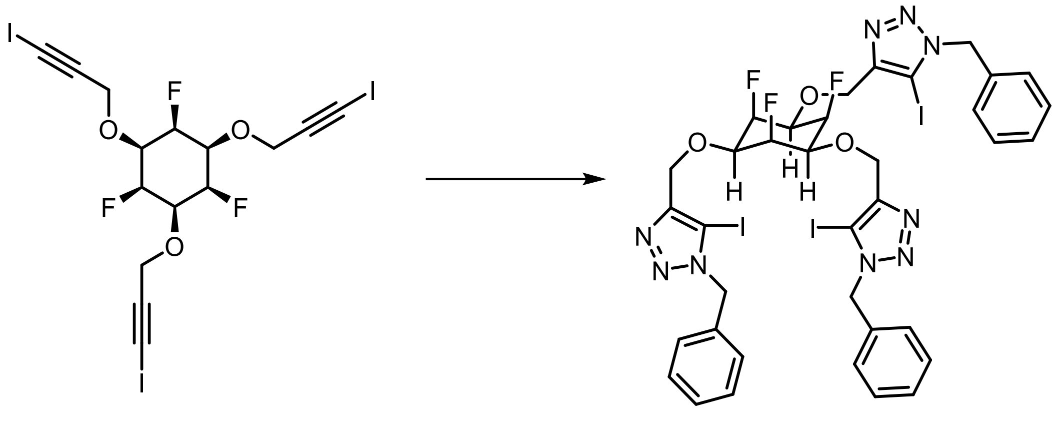

7 **9**

An oven dried Schlenk flask was charged with tetrakis acetonitrile copper hexafluorophosphate (63 mg, 0.17 mmol, 2.25 eq) and TBTA (90 mg, 0.17 mmol, 2.25 eq). The flask was evacuated and filled with nitrogen three times. Dry degassed THF (5 mL, degassed with nitrogen for fifteen minutes) was added to the flask and stirred for fifteen minutes. Another flask containing **8** (50 mg, 0.07 mmol, 1 eq) and benzyl azide (44.3 mg, 0.33 mmol, 4.5 eq) was dissolved in 2 mL THF and degassed with nitrogen for ten minutes and was added to the schlenk flask dropwise. The reaction was stirred over night. Ammonia solution (2 %- volume) was added and extracted with DCM. The combined organic layer was washed with brine and dried over $MgSO_4$. The solvent was evaporated under reduced pressure. Column chromatography was performed using 5:1 ethyl acetate and Acetone to give 9 as colourless solid (44 mg, 55%).

Melting point is 194 °C and colour change from white to yellow brown above this temperature.

^{1}H NMR (600 MHz, dichloromethane-d_2) δ 7.42 – 7.26 (m, 15H), 5.59 (s, 6H), 5.21 – 5.01 (m, 3H), 4.75 (s, 6H), 3.53 – 3.40 (m, 3H).

^{13}C NMR (151 MHz, dichloromethane-d_2) δ 148.32, 134.76, 129.31, 128.93, 128.33, 86.95 (d, split due to ^{13}C/^{19}F coupling, J = 193.9 Hz), 73.24, 62.30, 54.72.

^{19}F NMR (376 MHz, dichloromethane-d_2) δ -214.52 (s, 3F)

HR-MS (MALDI)- m/z = Cal. For $C_{36}H_{34}F_3I_3O_3N_9$: 1077.9871, observed: 1077.9868[M+H]$^+$

3. Supplementary figures

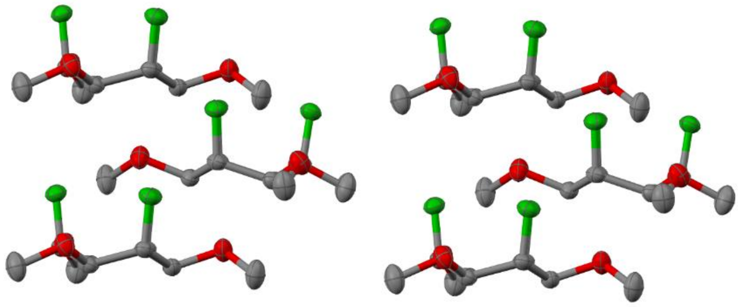

Fig. S1. Solid state packing of **2** (SCXRD). Stacks arrange such that all fluorine atoms point in the same direction in a non-centrosymmetric space group $P6_3$.

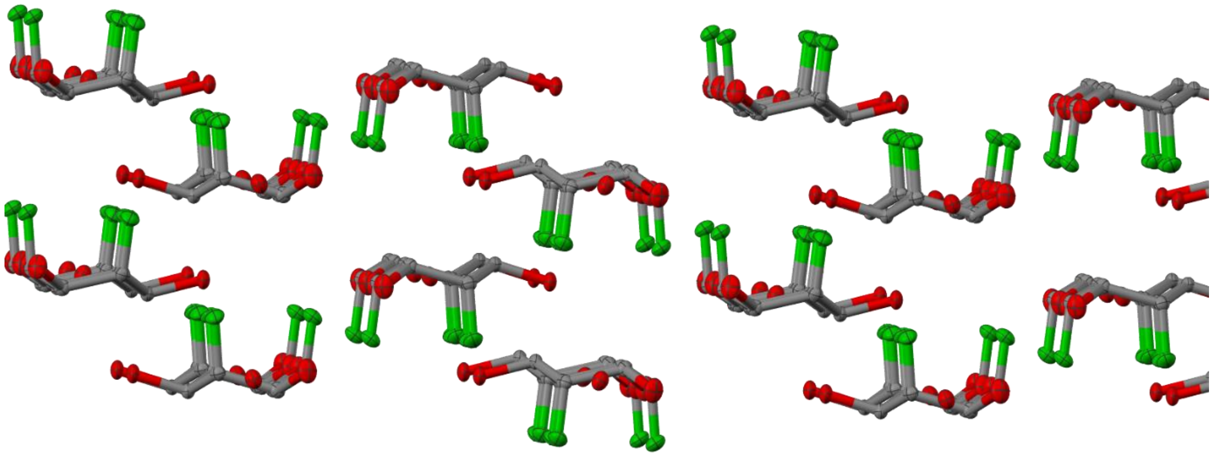

Fig. S2. Solid state packing of **3** (SCXRD). Stacks arrange such that half the fluorine atoms point in the opposite direction to cancel the macrodipole moment in a centrosymmetric space group Pnma.

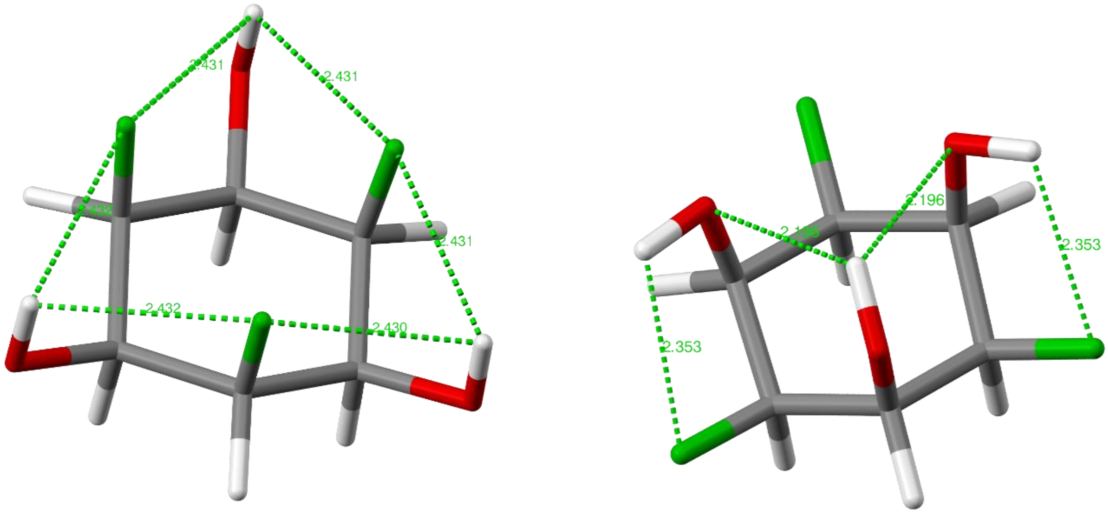

Fig. S3. Optimized geometry of **3** (DFT- PBE0/TZVP- D3, gas-phase) . F-axial (left) and F-equatorial (right) conformers. F-axial has six OH-F hydrogen bonds while F-equatorial has two OH-F hydrogen bonds and two OH-O hydrogen bonds.

4. DFT calculations

The DFT calculations were performed using Gaussian 16[7]. The input files for the DFT calculations were prepared using Avogadro. First the geometry of the conformers were optimized using the B3LYP-D3/def2-TZVP level of theory. The single point energies and molecular dipole moments were calculated over the geometry optimized structures using the Perdew-Burke-Ernzerhof hybrid functional (PBE0)[8] corrected with D3 empirical dispersion[9] and def2-TZVP basis set. We followed this particular computational approach, because this theoretical level was shown to compute the electronic energies of similar *Janus Face* compounds highly accurately by David O´Hagan and team[10, 11]. The difference in Gibbs free energy between the conformers was obtained by correcting the single point energy of the conformers obtained at the PBE0/def2-TZVP theoretical level with the B3LYP-D3/def2-TZVP thermal correction to free energy. The effect of solvents on the energy difference between the conformers were accounted for by the polarized continuum model[11]. The log files of the DFT calculations were uploaded to Zenodo: https://doi.org/10.5281/zenodo.10075518.

Table S1 Cartesian coordinates of the optimized geometries (using B3LYP-D3/def2-TZVP level) of F-axial and F-equatorial conformers of molecules **2** and **3** in gas-phase and in different solvents. The electronic energies (in Hartrees) were calculated at the PBE0-D3/def2-TZVP level of theory.

**2** F-axial (gas-phase)				**2** F-equatorial (gas-phase)			
Energy = -876.552999				Energy = -876.551499			
C	-1.15883	0.90967	-0.08357	C	1.36155	-0.91090	-0.41159
C	-1.34869	-0.54412	-0.53376	C	1.35869	0.48203	-1.07517
C	0.20302	1.43998	-0.53375	C	0.07725	-1.60974	-0.87322
H	-1.92548	1.55026	-0.52791	O	1.39917	-0.94131	0.98196
F	-1.30309	1.00473	1.28783	H	2.22233	-1.46021	-0.82635
C	1.36716	0.54871	-0.08345	C	-1.21593	-0.84441	-0.55025
O	0.28966	2.77259	-0.09699	F	0.00543	-2.88735	-0.34904
H	0.20388	1.40814	-1.63822	H	0.13112	-1.71718	-1.96328
C	1.14571	-0.89587	-0.53358	C	-1.09342	0.55877	-1.15809
F	1.52149	0.62625	1.28796	O	-1.46019	-0.81379	0.82996
H	2.30524	0.89245	-0.52773	H	-2.03405	-1.36527	-1.06562
C	-0.20840	-1.45824	-0.08356	C	0.15115	1.34331	-0.70432
H	-1.32160	-0.52749	-1.63825	H	1.34857	0.32892	-2.16087
O	-2.54601	-1.13549	-0.09691	F	2.53654	1.16438	-0.79268

H	-0.37975	-2.44245	-0.52796	H	0.18958	2.27059	-1.29714
F	-0.21875	-1.63086	1.28785	O	0.22909	1.64507	0.65972
O	2.25636	-1.63719	-0.09656	F	-2.25148	1.28815	-0.88246
H	1.11798	-0.88077	-1.63806	H	-1.04344	0.46385	-2.24967
C	2.29614	-2.98945	-0.51472	C	-2.82033	-0.97114	1.19825
C	-3.73705	-0.49360	-0.51456	C	2.58213	-0.45873	1.59845
C	1.44120	3.48291	-0.51460	C	-0.63222	2.66778	1.13177
H	1.59952	3.39425	-1.59804	H	-2.84674	-0.96347	2.28671
H	2.34227	3.14581	0.00717	H	-3.21953	-1.92870	0.84114
H	1.26611	4.52867	-0.26815	H	-3.44365	-0.15718	0.81599
H	3.28926	-3.36065	-0.26787	H	2.55751	-0.81985	2.62575
H	2.14088	-3.08181	-1.59830	H	2.61692	0.63191	1.59357
H	1.55347	-3.60159	0.00635	H	3.47990	-0.84825	1.10240
H	-3.73940	-0.31202	-1.59798	H	-0.61746	3.54019	0.46638
H	-3.89599	0.45515	0.00735	H	-0.25244	2.96013	2.10974
H	-4.55500	-1.16838	-0.26831	H	-1.66081	2.31641	1.23089

**2** F-axial (acetone)				**2** F-equatorial (acetone)			
Energy = -876.571758				Energy = -876.564854			
C	-1.44289	0.28482	-0.04851	C	1.44591	-0.69383	-0.46272
C	-0.95497	-1.09850	-0.49258	C	1.22687	0.70799	-1.05543
C	-0.47252	1.37606	-0.49085	C	0.26184	-1.55334	-0.91639
H	-2.42144	0.50196	-0.47935	O	1.55880	-0.74146	0.93625
F	-1.60927	0.30585	1.33842	H	2.35413	-1.10788	-0.92149
C	0.96947	1.10719	-0.04618	C	-1.12108	-1.00810	-0.54107
O	-0.97630	2.60954	-0.03192	F	0.40258	-2.84890	-0.42427
H	-0.46727	1.35569	-1.59146	H	0.30388	-1.62522	-2.00711
C	1.42943	-0.27850	-0.48976	C	-1.22291	0.41764	-1.10367
F	1.07040	1.23992	1.34113	O	-1.32788	-1.08119	0.84838
H	1.64702	1.84655	-0.47612	H	-1.86550	-1.62494	-1.05937
C	0.47477	-1.39347	-0.04836	C	-0.09740	1.36846	-0.66768
H	-0.94035	-1.08400	-1.59328	H	1.25379	0.61748	-2.14544
O	-1.77194	-2.15149	-0.03437	F	2.28442	1.54963	-0.70175
H	0.77728	-2.34915	-0.47927	H	-0.19462	2.29012	-1.25584

F	0.53688	-1.54920	1.33883	O	-0.09440	1.67806	0.70405
O	2.74878	-0.46060	-0.02942	F	-2.46013	0.96575	-0.74415
H	1.41125	-0.27095	-1.59038	H	-1.21293	0.35875	-2.19670
C	3.47122	-1.50595	-0.67096	C	-2.66473	-1.37342	1.23796
C	-3.04350	-2.24643	-0.66719	C	2.85003	-0.44582	1.45528
C	-0.42899	3.75890	-0.66884	C	-0.86712	2.81623	1.06731
H	-0.52309	3.68941	-1.75848	H	-2.65450	-1.48364	2.32121
H	0.62348	3.90927	-0.41082	H	-3.01031	-2.31136	0.78803
H	-1.00180	4.61317	-0.31348	H	-3.35226	-0.57009	0.96317
H	4.49660	-1.44018	-0.31255	H	2.83491	-0.73491	2.50516
H	3.46089	-1.38279	-1.75988	H	3.08657	0.61678	1.37510
H	3.07362	-2.49383	-0.42026	H	3.62501	-1.02369	0.93808
H	-2.94015	-2.28541	-1.75754	H	-0.57148	3.69552	0.48314
H	-3.69817	-1.41141	-0.40072	H	-0.66263	3.00523	2.12001
H	-3.49647	-3.17173	-0.31678	H	-1.93660	2.64133	0.93258

**2** F-axial (dichloromethane)				**2** F-equatorial (dichloromethane)			
Energy = -876.569203				Energy = -876.562988			
C	0.75352	-1.26363	-0.05479	C	1.46193	-0.67123	-0.45753
C	1.45561	0.02463	-0.49897	C	1.22296	0.72553	-1.05641
C	-0.70674	-1.27206	-0.49867	C	0.29234	-1.55066	-0.91176
H	1.24548	-2.13612	-0.48778	O	1.56853	-0.71517	0.94041
F	0.84938	-1.40281	1.32956	H	2.37851	-1.07167	-0.91288
C	-1.47181	-0.02069	-0.05487	C	-1.09915	-1.02274	-0.54193
O	-1.28339	-2.47257	-0.04035	F	0.44980	-2.84151	-0.41620
H	-0.69520	-1.25392	-1.59988	H	0.33853	-1.62462	-2.00254
C	-0.74841	1.24810	-0.49865	C	-1.21776	0.39736	-1.11424
F	-1.64115	-0.03481	1.32943	O	-1.30568	-1.09066	0.84709
H	-2.47353	-0.01148	-0.48803	H	-1.83402	-1.65362	-1.05789
C	0.71834	1.28522	-0.05476	C	-0.11096	1.36719	-0.66982
H	1.43453	0.02484	-1.60028	H	1.24803	0.62990	-2.14635
O	2.78427	0.12610	-0.04212	F	2.26999	1.58200	-0.71115
H	1.22732	2.14765	-0.48880	H	-0.22229	2.28896	-1.25700
F	0.79124	1.43820	1.32988	O	-0.10571	1.67488	0.70063

O	-1.50098	2.34735	-0.04177	F	-2.46973	0.92788	-0.78175
H	-0.73821	1.22916	-1.59995	H	-1.18909	0.33150	-2.20696
C	-1.17457	3.59498	-0.64181	C	-2.64170	-1.38242	1.23818
C	3.70057	-0.78198	-0.64124	C	2.84389	-0.37577	1.46990
C	-2.52578	-2.81514	-0.64221	C	-0.96359	2.73963	1.09159
H	-2.45289	-2.80458	-1.73588	H	-2.63126	-1.48360	2.32221
H	-3.33151	-2.14369	-0.33140	H	-2.98486	-2.32514	0.79581
H	-2.76399	-3.82361	-0.30988	H	-3.33160	-0.58341	0.95670
H	-1.93183	4.30457	-0.31429	H	2.83219	-0.67389	2.51722
H	-1.19514	3.52614	-1.73566	H	3.04105	0.69554	1.39973
H	-0.19249	3.95852	-0.32534	H	3.64347	-0.92017	0.95330
H	3.65411	-0.72760	-1.73495	H	-0.79899	3.62750	0.46944
H	3.52081	-1.81443	-0.32725	H	-0.70869	2.97775	2.12311
H	4.69369	-0.48430	-0.31071	H	-2.01635	2.45596	1.03499

**3** F-axial (gas-phase)				**3** F-equatorial (gas-phase)			
Energy = -758.780929				Energy = -758.773067			
C	0.62833	-1.33010	-0.71004	C	-1.28974	0.73986	-0.25014
C	1.46684	-0.12100	-0.29807	C	-1.23358	-0.71490	-0.72948
C	-0.83820	-1.20975	-0.29812	C	0.00000	1.43921	-0.67879
O	1.19721	-2.53442	-0.24792	O	-1.45008	0.81925	1.15273
H	0.65197	-1.38009	-1.80386	H	-2.12490	1.24297	-0.75443
C	-1.46604	0.12098	-0.71018	C	1.28974	0.73986	-0.25014
F	-0.93389	-1.34757	1.09382	F	0.00000	2.75145	-0.24437
H	-1.41005	-2.03524	-0.72620	H	0.00000	1.46336	-1.77574
C	-0.62861	1.33078	-0.29804	C	1.23358	-0.71490	-0.72948
O	-2.79343	0.23049	-0.24802	O	1.45008	0.81925	1.15273
H	-1.52106	0.12553	-1.80399	H	2.12491	1.24297	-0.75443
C	0.83778	1.20917	-0.71004	C	-0.00000	-1.47405	-0.21607
H	2.46767	-0.20357	-0.72613	H	-1.24723	-0.74076	-1.82470
F	1.63396	-0.13476	1.09387	F	-2.39415	-1.35374	-0.29401
H	0.86916	1.25460	-1.80386	H	-0.00000	-2.47147	-0.66146
O	1.59639	2.30395	-0.24797	O	-0.00000	-1.67575	1.17380
F	-0.70037	1.48225	1.09394	F	2.39415	-1.35374	-0.29401

H	-1.05742	2.23890	-0.72599	H	1.24723	-0.74077	-1.82470
H	1.18424	-2.50715	0.71791	H	-2.23879	0.31268	1.38430
H	-2.76313	0.22815	0.71780	H	2.23879	0.31268	1.38430
H	1.57956	2.27894	0.71787	H	0.00000	-0.81009	1.60899

**3** F-axial (methanol)				**3** F-equatorial (methanol)			
Energy = -758.794164				Energy = -758.789509			
C	-0.24069	-1.44972	-0.71372	C	-1.28873	0.73957	-0.25736
C	1.13670	-0.93488	-0.30311	C	-1.23472	-0.71636	-0.73102
C	-1.37787	-0.51633	-0.30489	C	-0.00001	1.43318	-0.69311
O	-0.46031	-2.76558	-0.24296	O	-1.43869	0.82893	1.15179
H	-0.24862	-1.50723	-1.80547	H	-2.12658	1.23847	-0.75628
C	-1.13451	0.93450	-0.71375	C	1.28872	0.73959	-0.25736
F	-1.53798	-0.57607	1.09429	F	-0.00002	2.75155	-0.24158
H	-2.31742	-0.86859	-0.73194	H	-0.00001	1.46913	-1.78619
C	0.24177	1.45159	-0.30227	C	1.23473	-0.71634	-0.73102
O	-2.16510	1.78180	-0.24334	O	1.43867	0.82895	1.15179
H	-1.17805	0.97255	-1.80547	H	2.12656	1.23851	-0.75628
C	1.37604	0.51609	-0.71355	C	0.00001	-1.47706	-0.22938
H	1.91206	-1.57268	-0.72891	H	-1.26189	-0.74665	-1.82323
F	1.26730	-1.04235	1.09620	F	-2.39142	-1.35982	-0.27454
H	1.42837	0.53606	-1.80547	H	0.00002	-2.46967	-0.68310
O	2.62577	0.98463	-0.24452	O	0.00002	-1.69060	1.16700
F	0.26918	1.61655	1.09735	F	2.39145	-1.35978	-0.27454
H	0.40703	2.44245	-0.72670	H	1.26190	-0.74663	-1.82323
H	-0.45710	-2.73559	0.72318	H	-2.27219	0.40070	1.38531
H	-2.14312	1.76139	0.72279	H	2.27218	0.40074	1.38532
H	2.59877	0.97413	0.72167	H	0.00001	-0.82592	1.60450

**3** F-axial (dichloromethane)				**3** F-equatorial (dichloromethane)			
Energy = -758.792338				Energy = -758.787048			
C	-1.21597	0.82588	-0.71313	C	-1.28883	0.73963	-0.25524
C	-1.32458	-0.64073	-0.30241	C	-1.23429	-0.71589	-0.73047

C	0.10709	1.46758	-0.30162	C	-0.00002	1.43369	-0.69069
O	-2.31975	1.57538	-0.24497	O	-1.43983	0.82771	1.15273
H	-1.26211	0.85802	-1.80524	H	-2.12628	1.23921	-0.75501
C	1.32270	0.64018	-0.71284	C	1.28881	0.73967	-0.25524
F	0.11950	1.63411	1.09701	F	-0.00004	2.75178	-0.24384
H	0.18048	2.46945	-0.72636	H	-0.00002	1.46612	-1.78443
C	1.21774	-0.82638	-0.30114	C	1.23431	-0.71585	-0.73047
O	2.52368	1.22268	-0.24646	O	1.43979	0.82775	1.15274
H	1.37227	0.66226	-1.80515	H	2.12624	1.23928	-0.75501
C	-0.10666	-1.46502	-0.71274	C	0.00002	-1.47672	-0.22692
H	-2.22792	-1.07848	-0.72897	H	-1.25843	-0.74459	-1.82323
F	-1.47824	-0.71454	1.09577	F	-2.39206	-1.35897	-0.27889
H	-0.10980	-1.51851	-1.80516	H	0.00004	-2.46981	-0.67990
O	-0.20443	-2.79637	-0.24676	O	0.00003	-1.68911	1.16826
F	1.35905	-0.92254	1.09689	F	2.39211	-1.35889	-0.27889
H	2.04829	-1.39011	-0.72782	H	1.25845	-0.74455	-1.82323
H	-2.29604	1.55823	0.72120	H	-2.26709	0.38787	1.38677
H	2.49792	1.21181	0.71976	H	2.26707	0.38794	1.38678
H	-0.20398	-2.76863	0.71948	H	0.00002	-0.82455	1.60605

5. NMR host-guest titrations

Tetrabutylammonium chloride (TBACl) and host molecules **8** and **9** were dried under high vacuum over phosphorus pentoxide overnight in a round bottom flask. NMR solvents were dried by adding molecular sieves (3 Å) and kept overnight before usage. ^{19}F NMR was calibrated to hexafluorobenzene (-164.9 ppm), which was added as an internal standard. Titrations were performed by addition of aliquots of TBACl solution (100-300 mM) dissolved in the host solution (to nullify dilution effect) to the host solution. The titration data was fitted using online tool Bindfit (http://supramolecular.org). All titrations were performed in triplicate to calculate the standard error in the experiments.

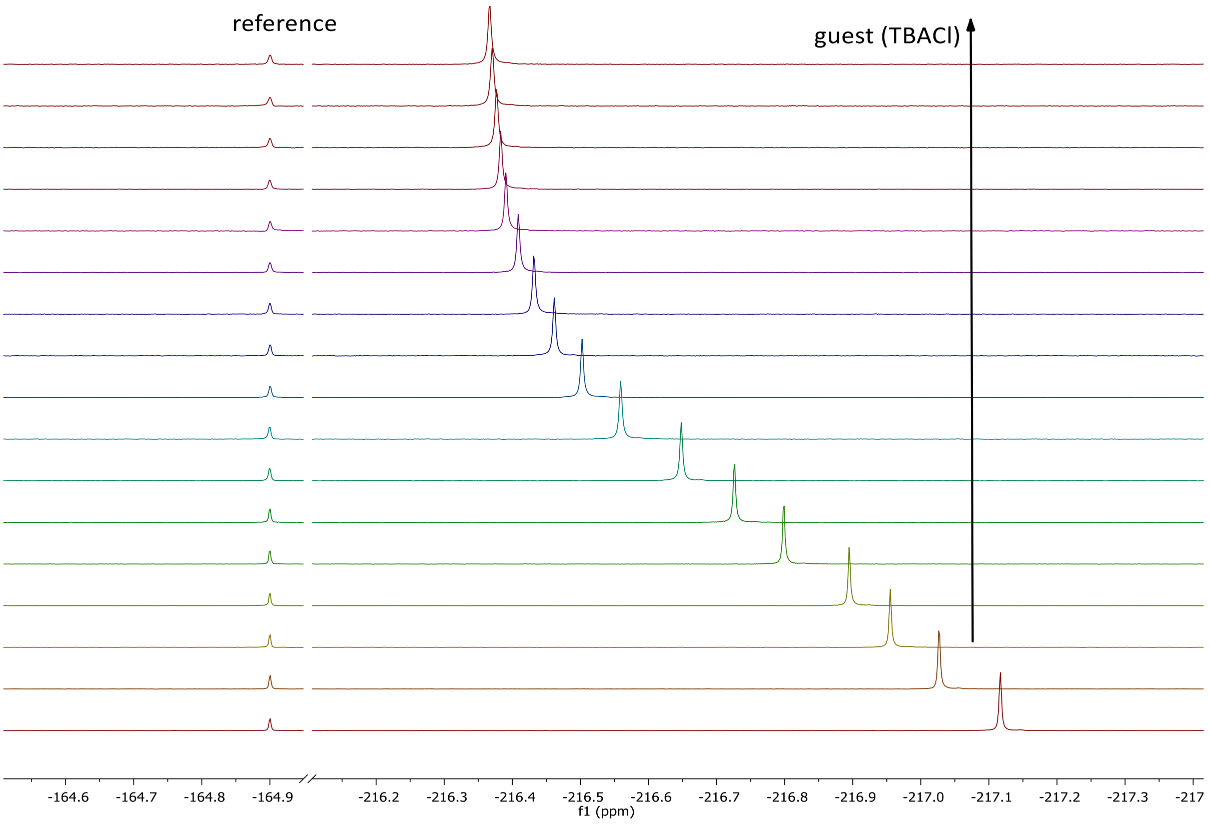

Fig. S4. ^{19}F NMR titration of **8** with TBACl in CD_2Cl_2 at 298K.

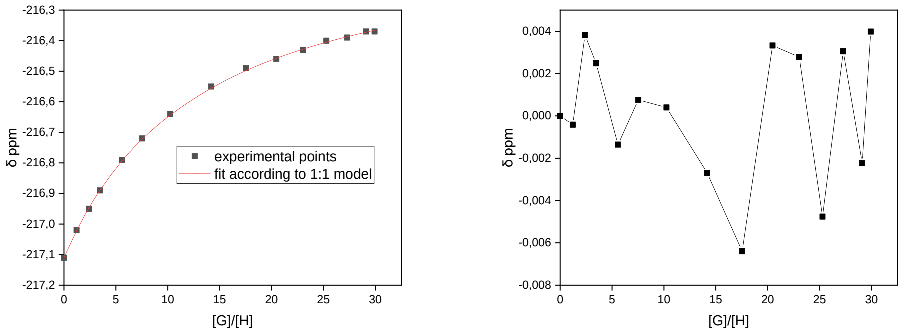

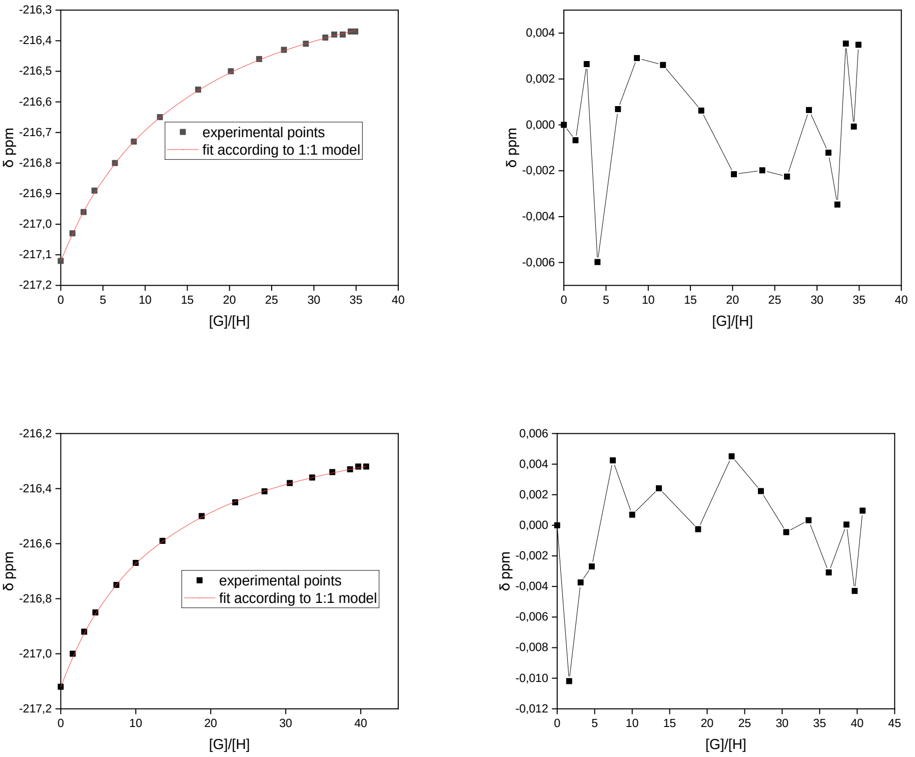

Fig. S5. Binding isotherms for titration (^{19}F NMR, 298 K) of **8** with TBACl in CD_2Cl_2 and the corresponding residual plots and the link to Supramolecular.org.

titration 1

http://app.supramolecular.org/bindfit/view/ccdc3b44-e1f8-4de4-8eb4-f641d8b1467b

titration 2

http://app.supramolecular.org/bindfit/view/ffffd7f7-acf9-497c-a364-fdbd97d4faa1

titration 3

http://app.supramolecular.org/bindfit/view/7c87c380-8249-44ed-9af9-69d4ff0e12e9

Table S2. Association constants obtained for the titration of **8** with TBACl in CD_2Cl_2 at 298 K. Where n = 3 (number of trials performed), s is the sample standard deviation, $t_{(0.05,\ 2)}* s/\sqrt{n}$- 95% confidence interval and $t_{(0.05,\ 2)}$ is the Student´s t-value at 95% confidence level[12].

Entry	K_a (M^{-1})	Fit error	Mean K_a (M^{-1})	$s/\sqrt{n}$ (M^{-1})	$t_{(0.05,\ 2)}* s/\sqrt{n}$ (M^{-1})
1	29	±0.9%	28.7	0.3	±1.4
2	29	±0.7%			
3	28	±1%			

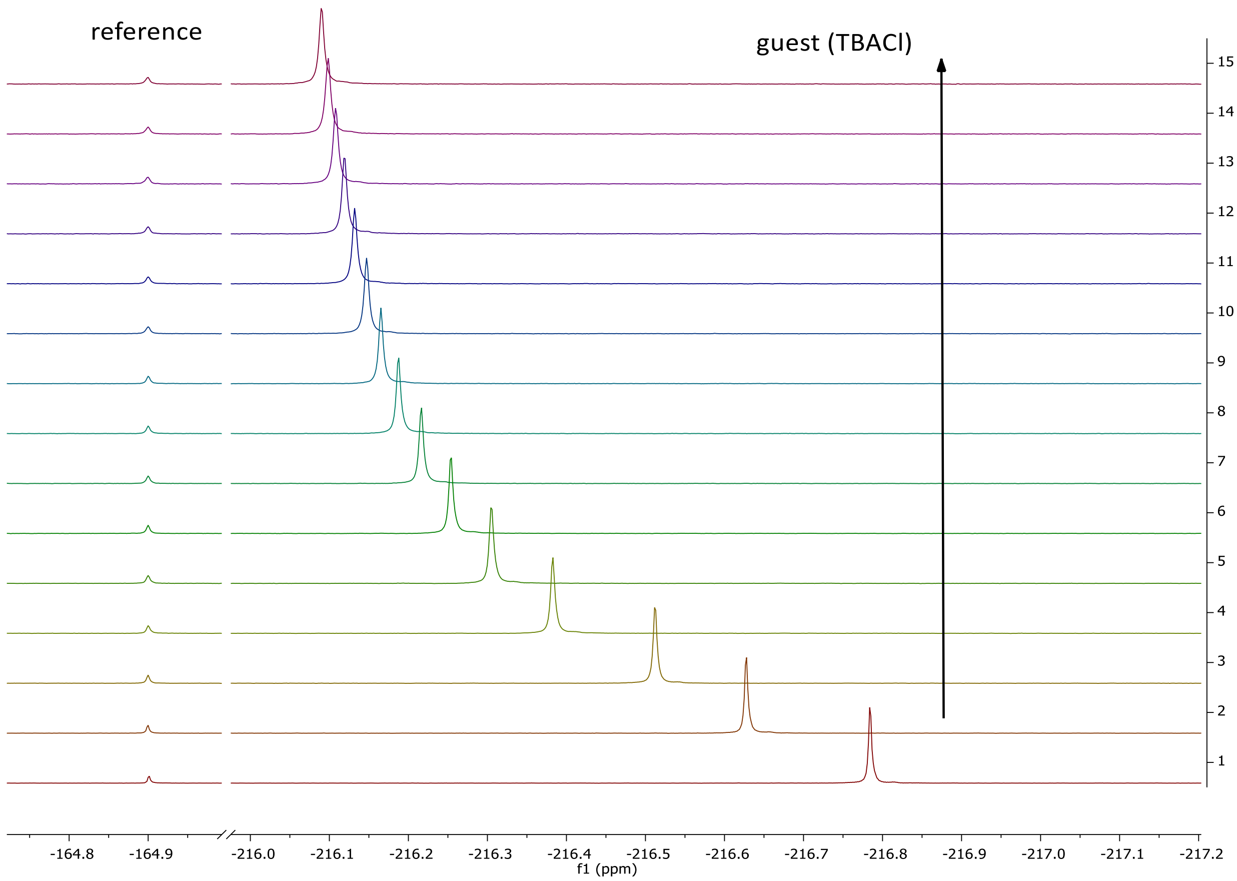
reference
guest (TBACl)
15
14
13
12
11
10
9
8
7
6
5
4
3
2
1
-164.8
-164.9
-216.0
-216.1
-216.2
-216.3
-216.4
-216.5
-216.6
-216.7
-216.8
-216.9
-217.0
-217.1
-217.2
f1 (ppm)

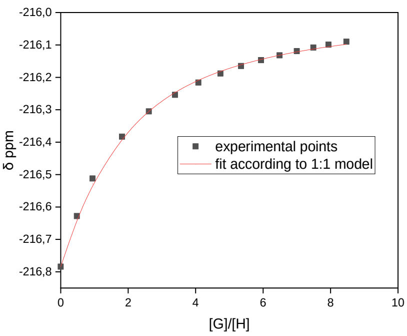
δ ppm
[G]/[H]
experimental points
fit according to 1:1 model

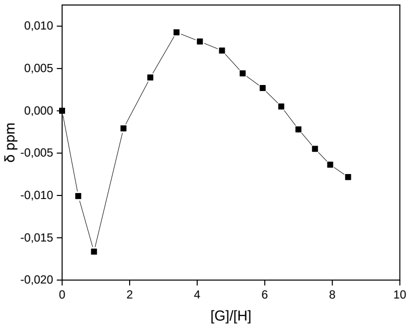
δ ppm
[G]/[H]

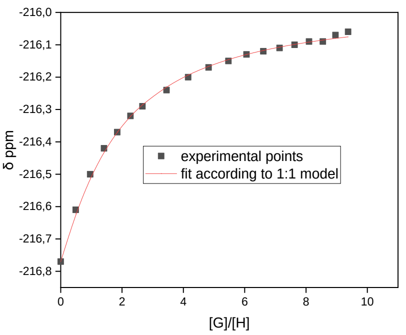
δ ppm
[G]/[H]
experimental points
fit according to 1:1 model

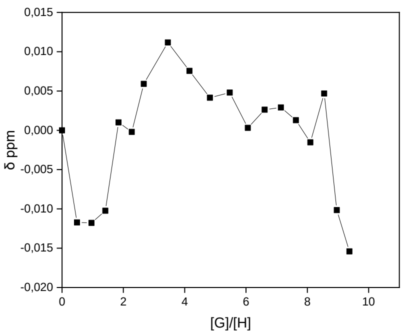
δ ppm
[G]/[H]

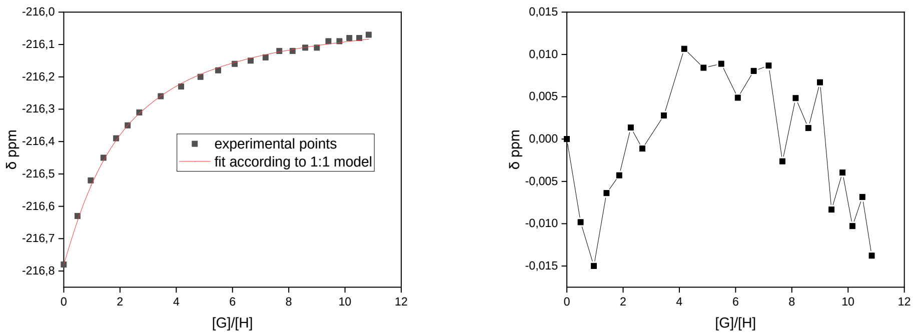

Fig. S6. Binding isotherms for titration (^{19}F NMR, 298 K) of **9** with TBACl in CD_2Cl_2 and the corresponding residual plots and the link to Supramolceular.org.

titration 1

http://app.supramolecular.org/bindfit/view/6b957fd5-4c12-4961-911f-193a12a5a2d4

titration 2

http://app.supramolecular.org/bindfit/view/326fc54c-1cd1-48cc-9a20-20809fbc1af0

titration 3

http://app.supramolecular.org/bindfit/view/c88104a0-0cda-4291-b48e-b498d5b7651b

Table S3. Association constants obtained for the titration of **9** with TBACl in CD_2Cl_2 at 298 K.

Entry	K_a (M^{-1})	Fit error	Mean K_a (M^{-1})	$s/\sqrt{n}$ (M^{-1})	$t_{(0.05,\,2)}{*}\,s/\sqrt{n}$ (M^{-1})
1	150	±3%	142	5	±23
2	144	±3%			
3	132	±3%			

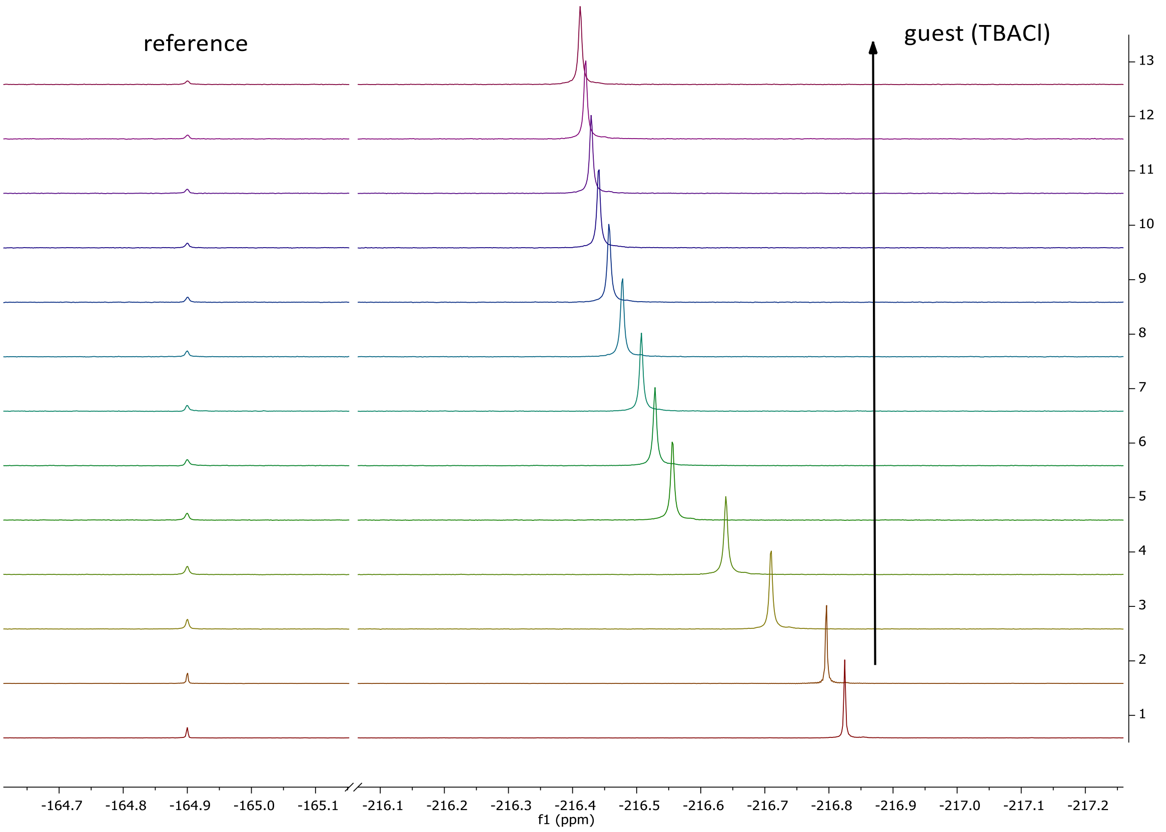

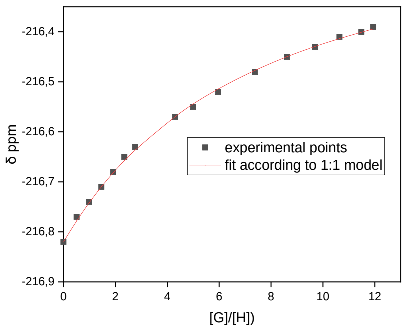

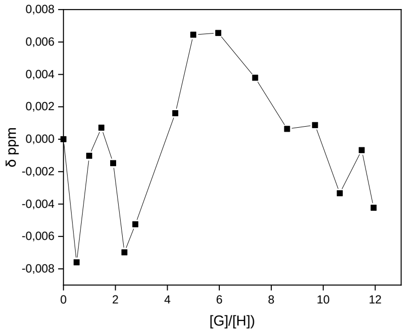

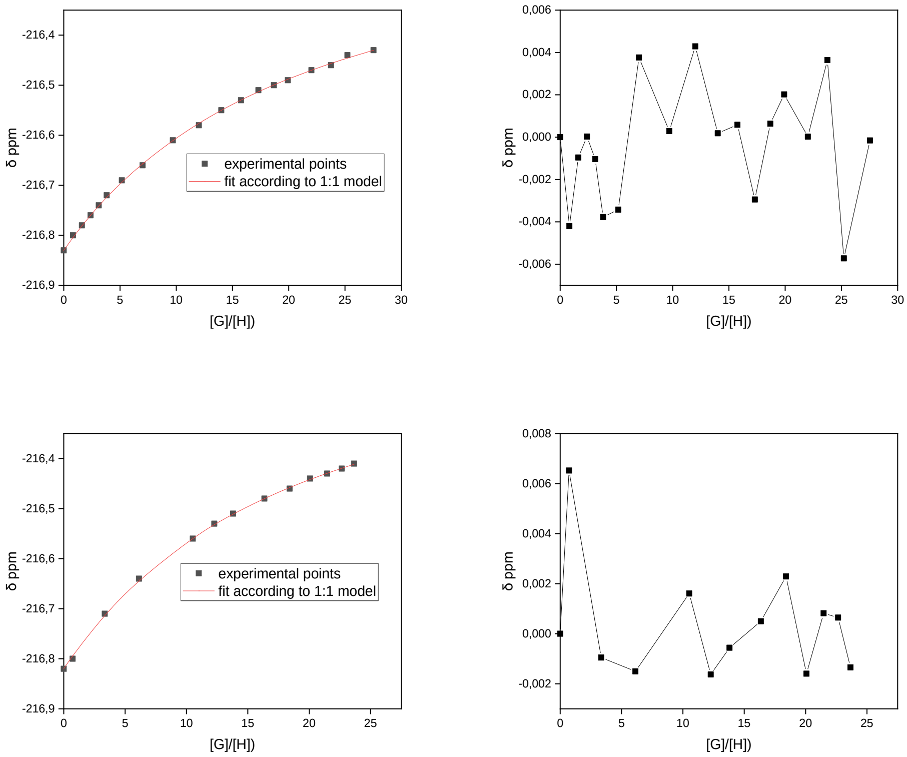

Fig. S7. Binding isotherms for titration (^{19}F NMR, 298 K) of **2** with TBACl in CD_2Cl_2 at 298 K and the corresponding residual plots and the links to Supramolecular.org.

titration 1

http://app.supramolecular.org/bindfit/view/26b0ac62-bba0-4d8e-bc21-cdcba093e773

titration 2

http://app.supramolecular.org/bindfit/view/914ae70b-26c3-4c96-8840-13ca20aaaa72

titration 3

http://app.supramolecular.org/bindfit/view/a049bc8c-fe02-4fde-8f22-a98080b3118f

Table S4. Association constant obtained for the titration of **2** with TBACl in CD_2Cl_2 at 298 K.

Entry	K_a (M^{-1})	Fit error	Mean K_a (M^{-1})	$s/\sqrt{n}$ (M^{-1})	$t_{(0.05, 2)}$* $s/\sqrt{n}$ (M^{-1})
1	16	±2%	15	0.6	±3
2	15.5	±1%			
3	14	±1%			

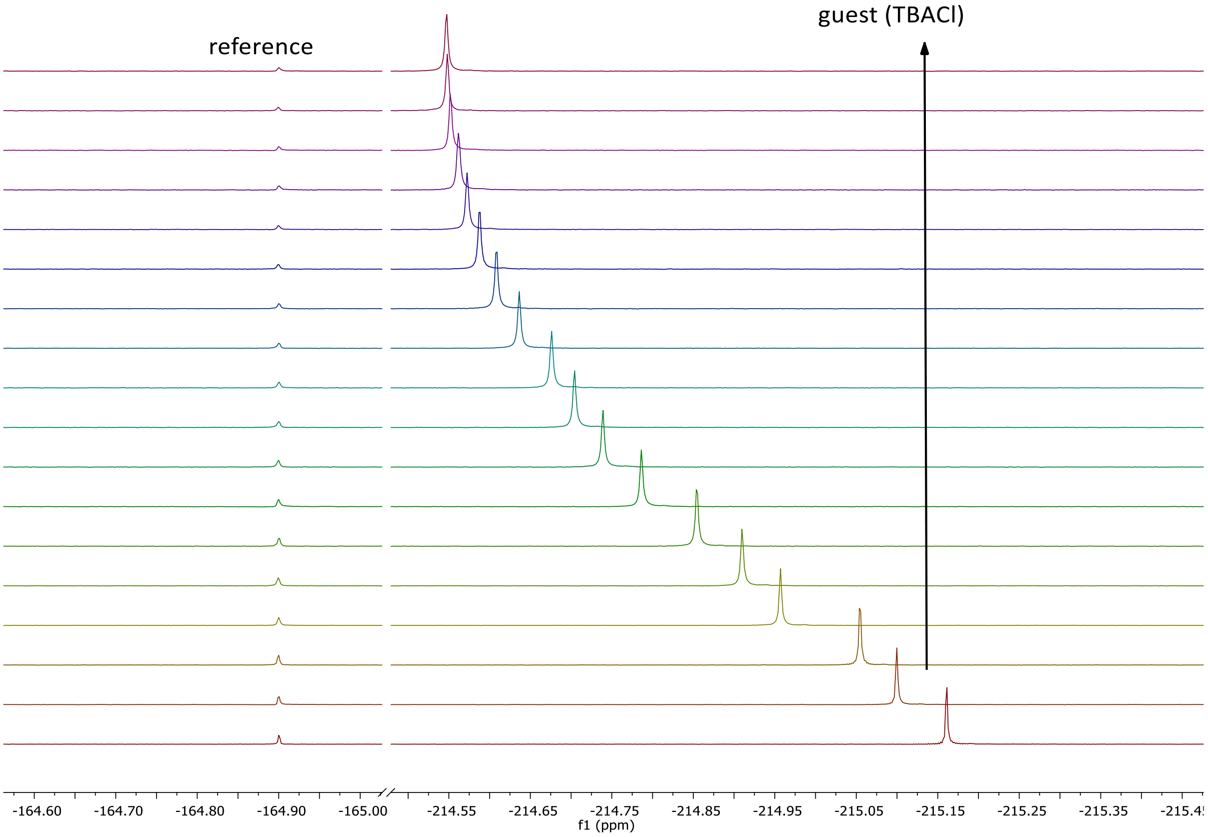

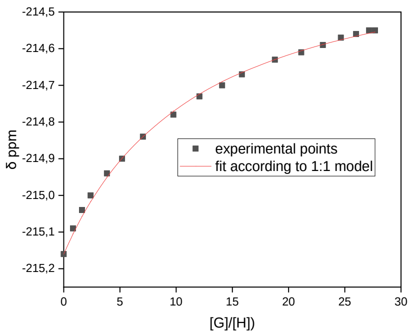

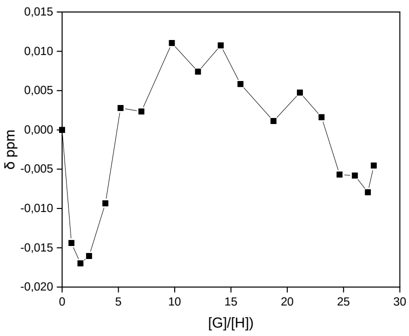

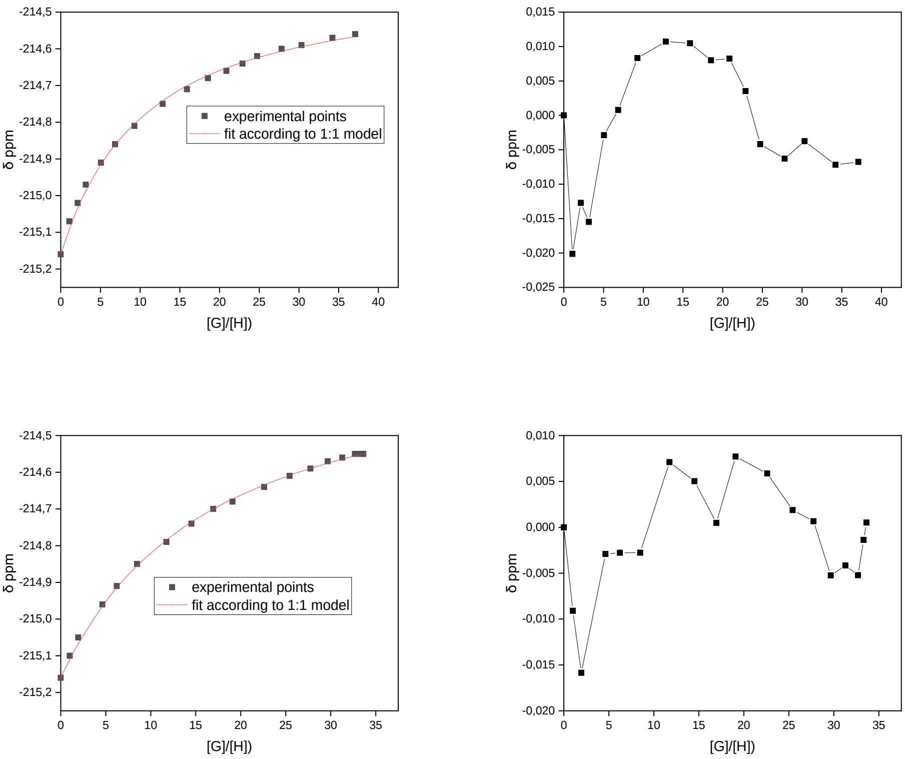

Fig. S8. Binding isotherms for titration (^{19}F NMR, 298 K) of **2** with TBACl in acetone-d_6 at 298 K and the corresponding residual plots and the links to Supramolecular.org.

titration 1

http://app.supramolecular.org/bindfit/view/e7d68cec-9735-434c-a00c-043e31523001

titration 2

http://app.supramolecular.org/bindfit/view/6920e0e9-2518-4bd8-b9d5-50492ce1ddbe

titration 3

http://app.supramolecular.org/bindfit/view/8dd4d7ff-4c11-47a8-ad99-ca55576bc9b7

Table S5. Association constant obtained for the titration of **2** with TBACl in acetone at 298 K.

Entry	K_a (M^{-1})	Fit error	Mean K_a (M^{-1})	$s/\sqrt{n}$ (M^{-1})	$t_{(0.05, 2)}$* $s/\sqrt{n}$ (M^{-1})
1	30	±3%	25	4	±16
2	28	±4%			
3	18	±2%			

6. VT-NMR experiment

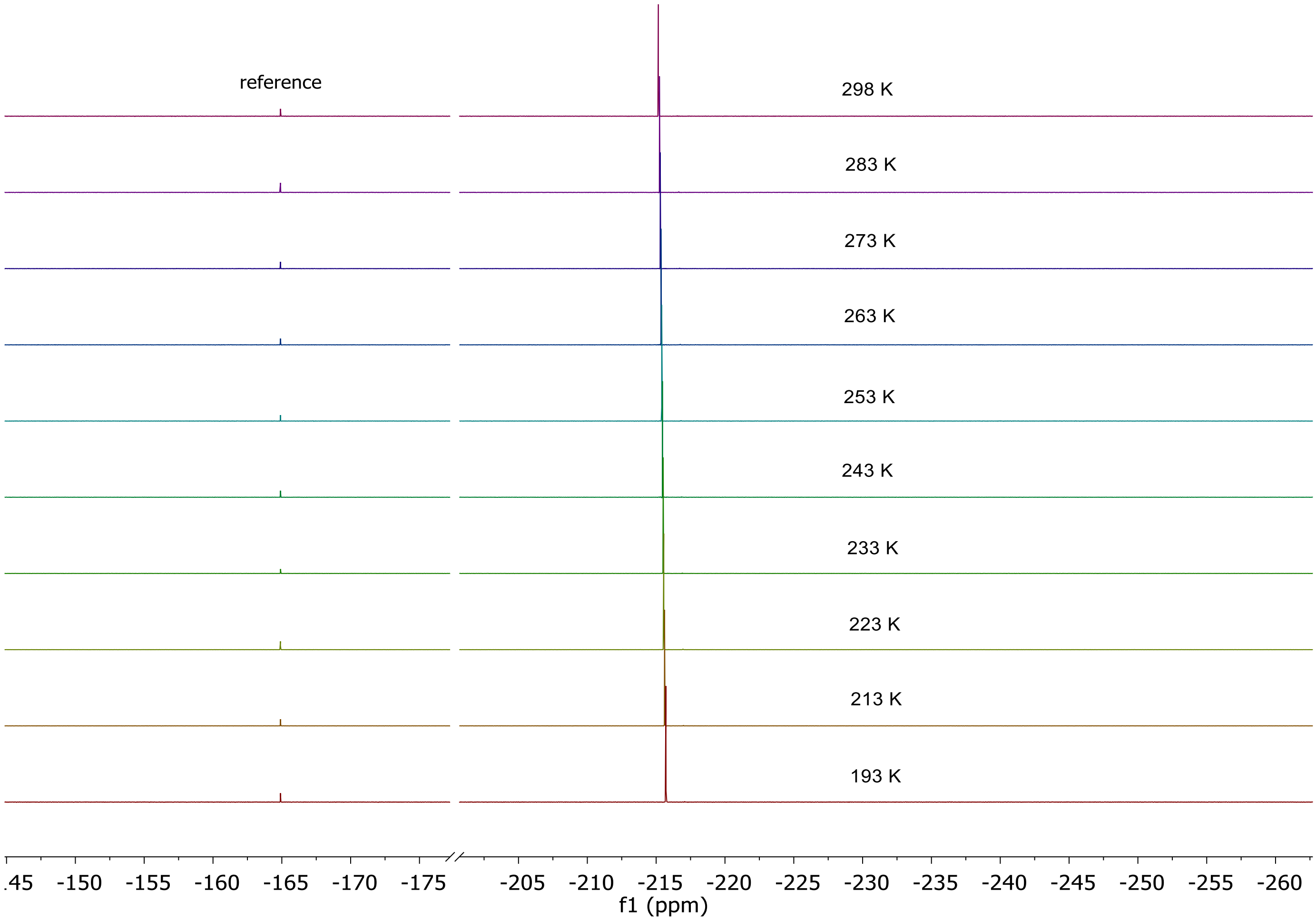

Fig. S9. VT- ^{19}F NMR of **2** in acetone-d_6; only one peak (corresponding to the major conformer) was observed over the whole temperature range.

7. Characterization data

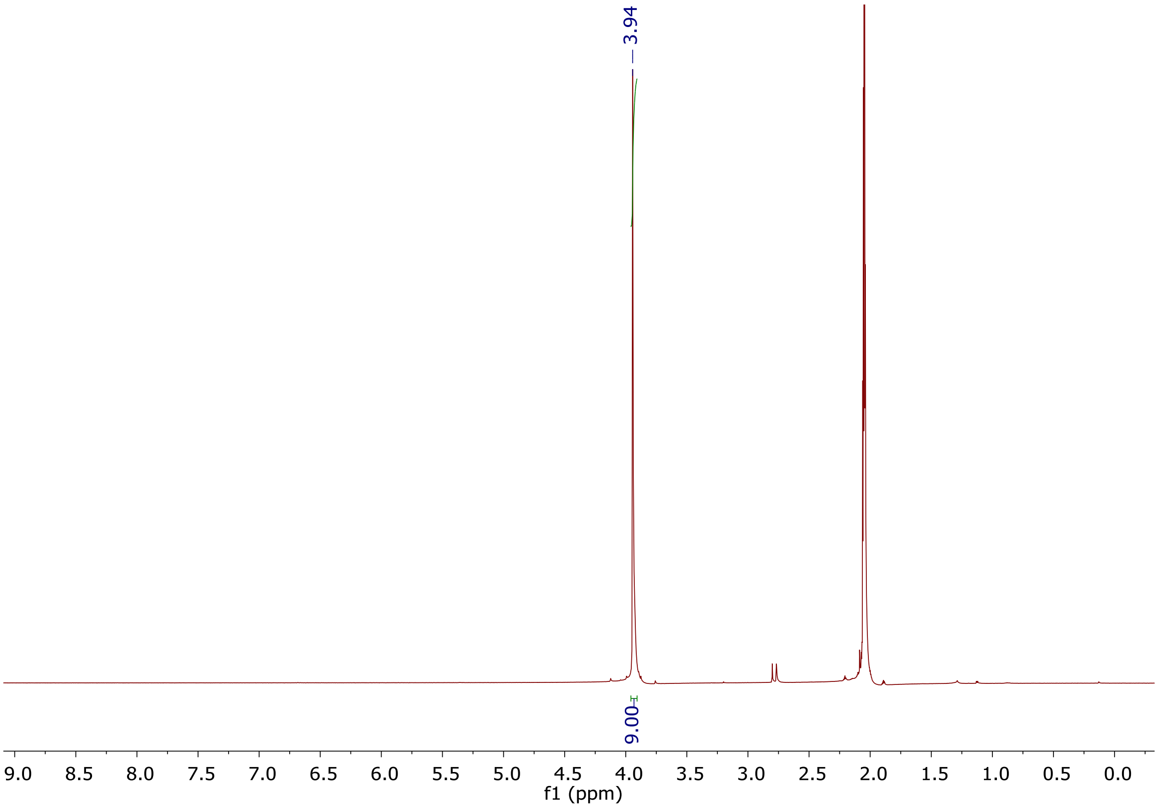

Fig. S10 ^{1}H NMR (400 MHz, 298 K, acetone-d_6) of **S2**

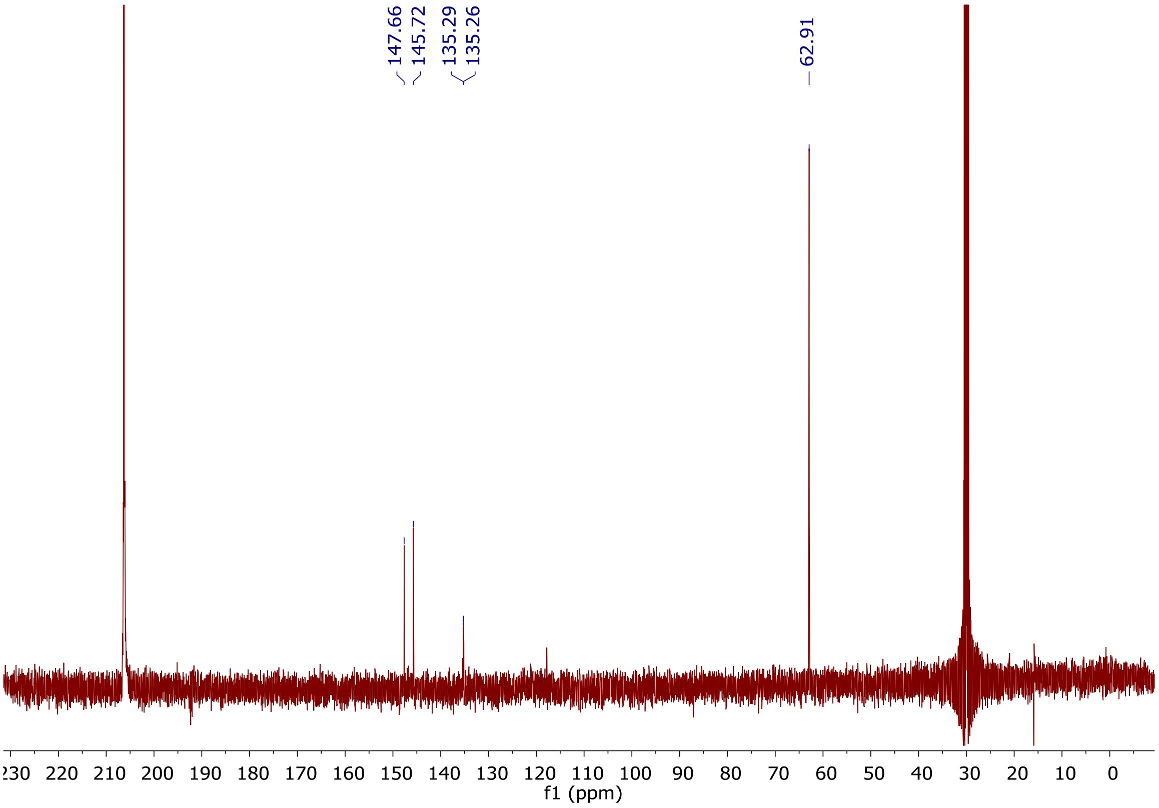

Fig. S11 ^{13}C NMR (126 MHz, 298 K, acetone-d_6) of **S2**

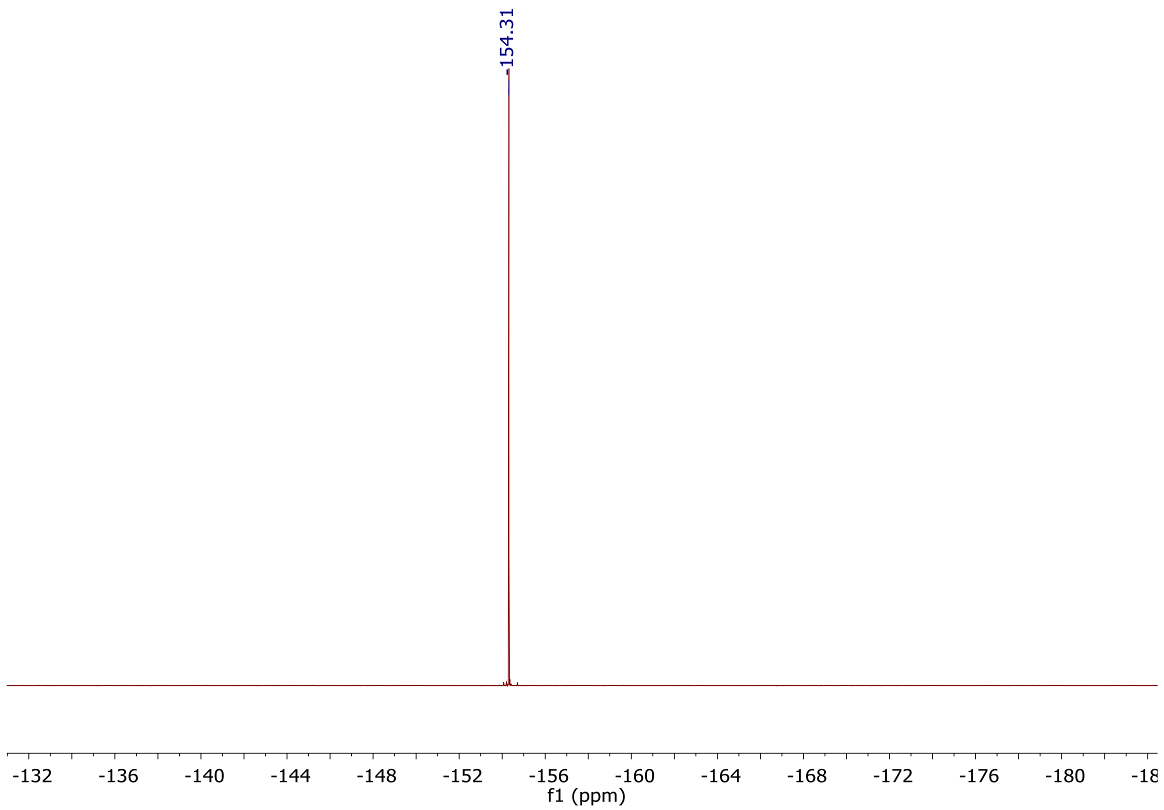

Fig. S12 ^{19}F NMR (376 MHz, 298 K, acetone-d_6) of **S2**

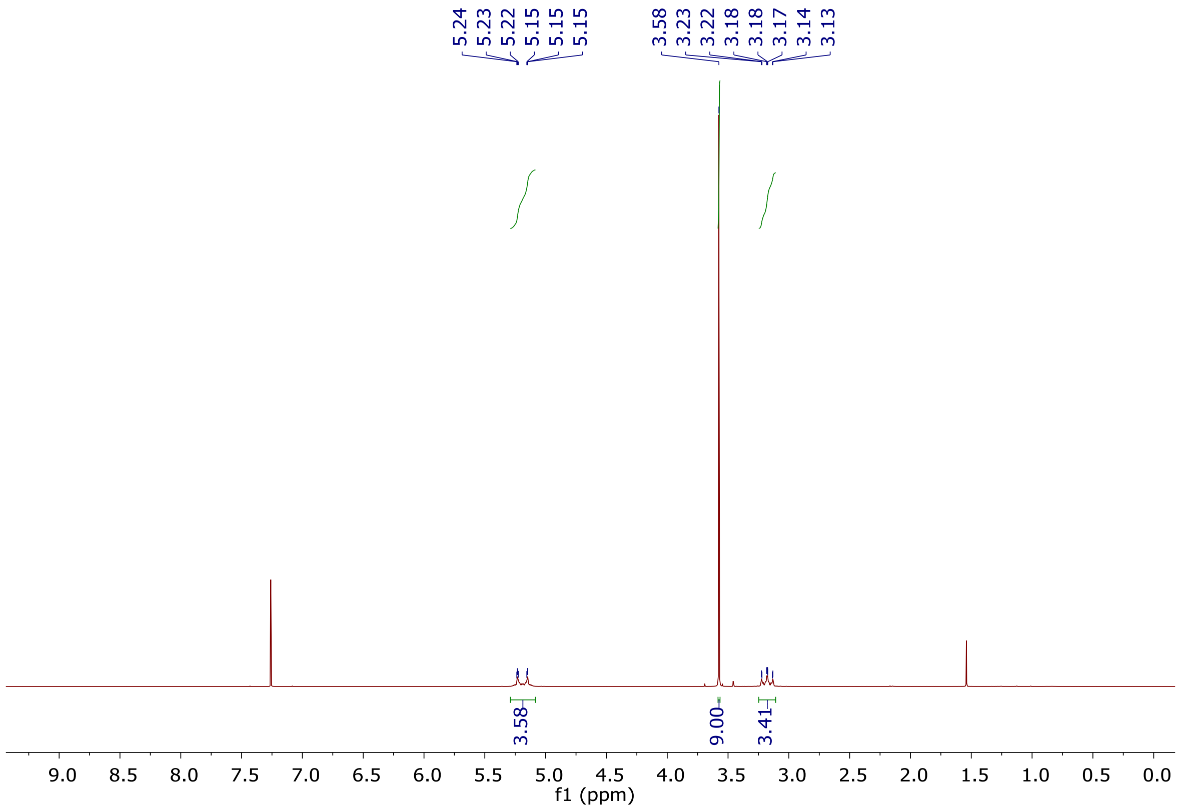

Fig. S13 ^{1}H NMR (600 MHz, 298 K, chloroform-d) of **2**

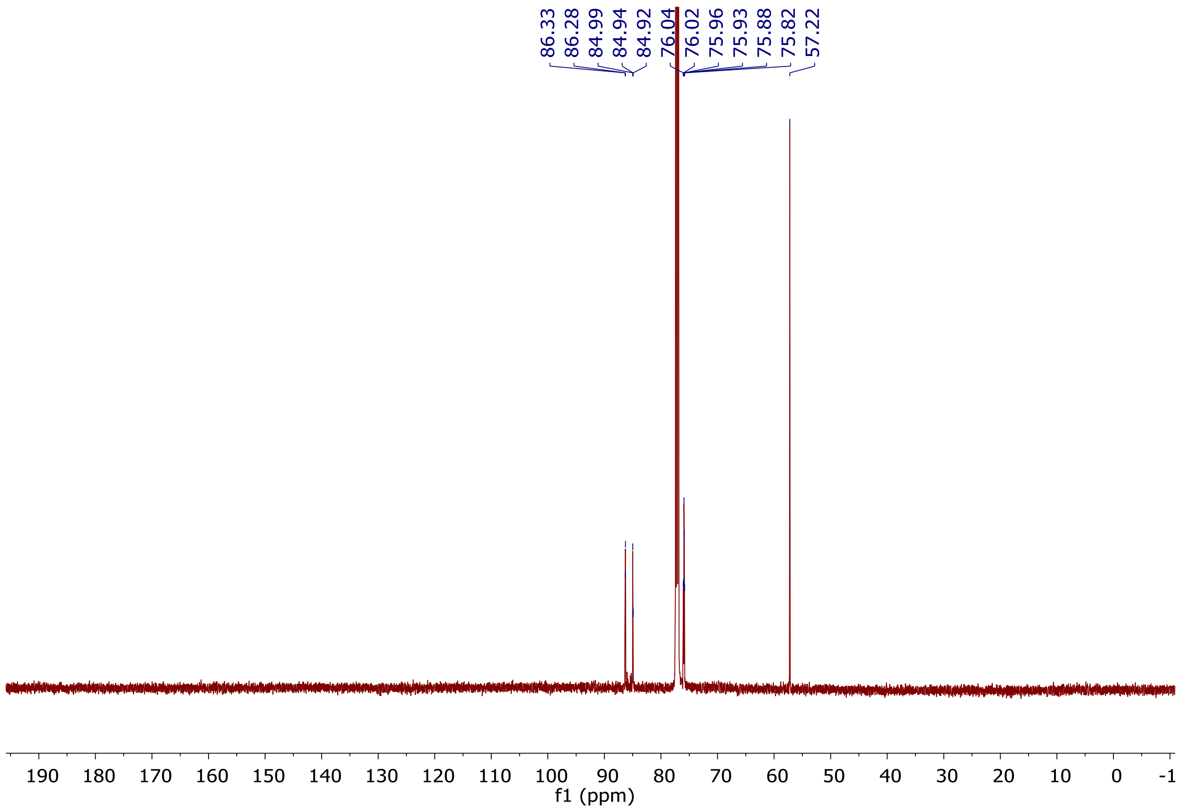

Fig. S14 ^{13}C NMR (151 MHz, 298 K, chloroform-*d*) of **2**

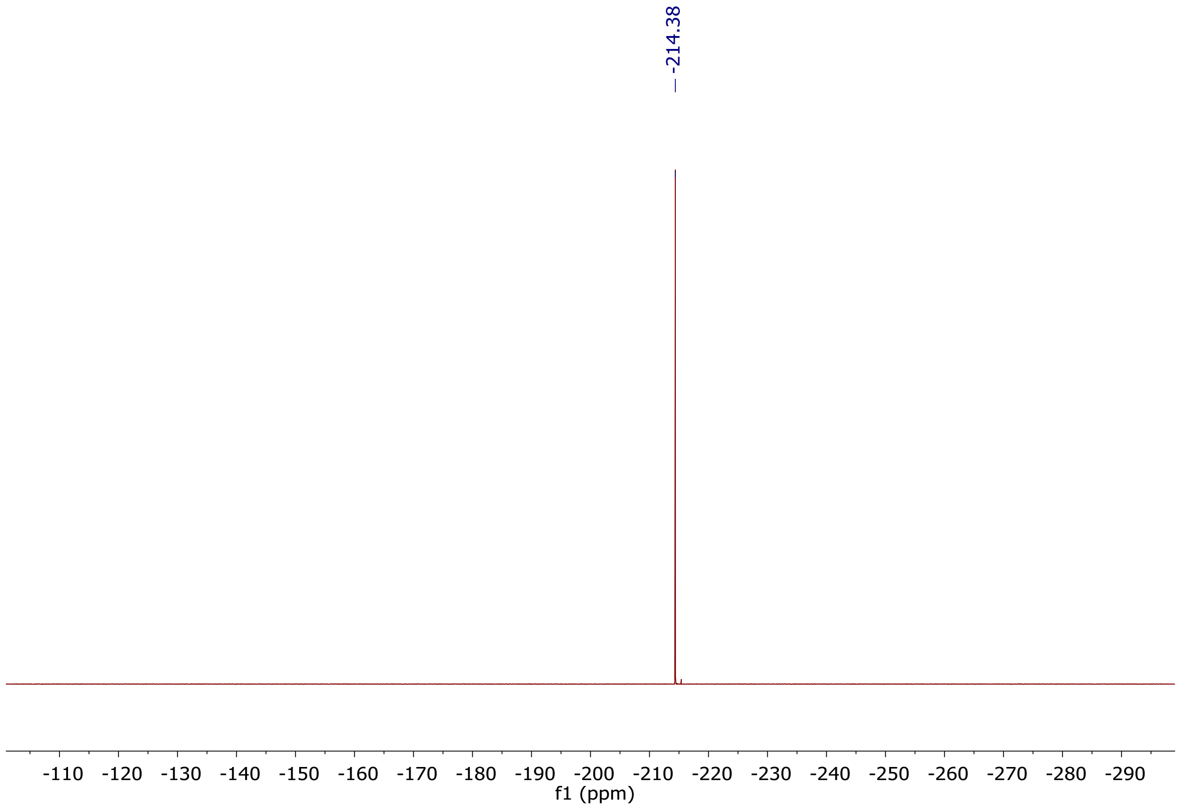

Fig. S15 ^{19}F NMR (376 MHz, 298 K, chloroform-*d*) of **2**

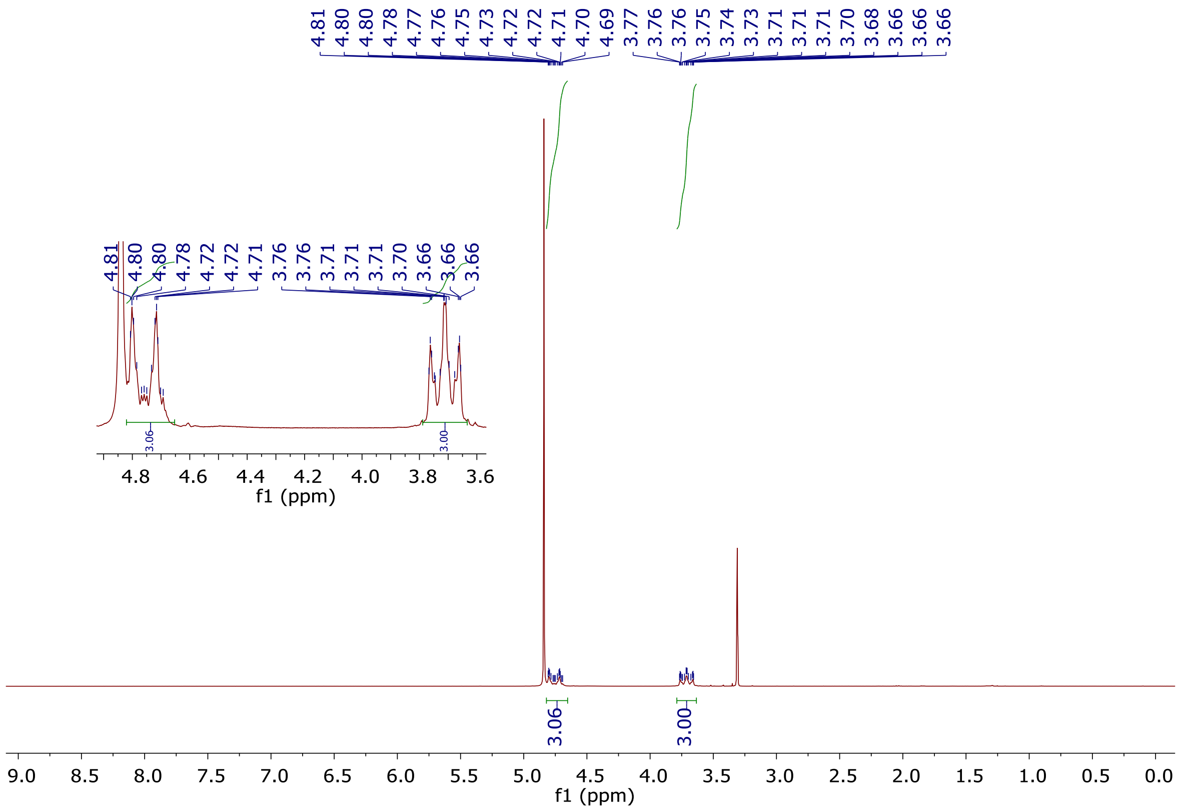

Fig. S16 ^{1}H NMR (600 MHz, 298 K, methanol-d_4) of **3**

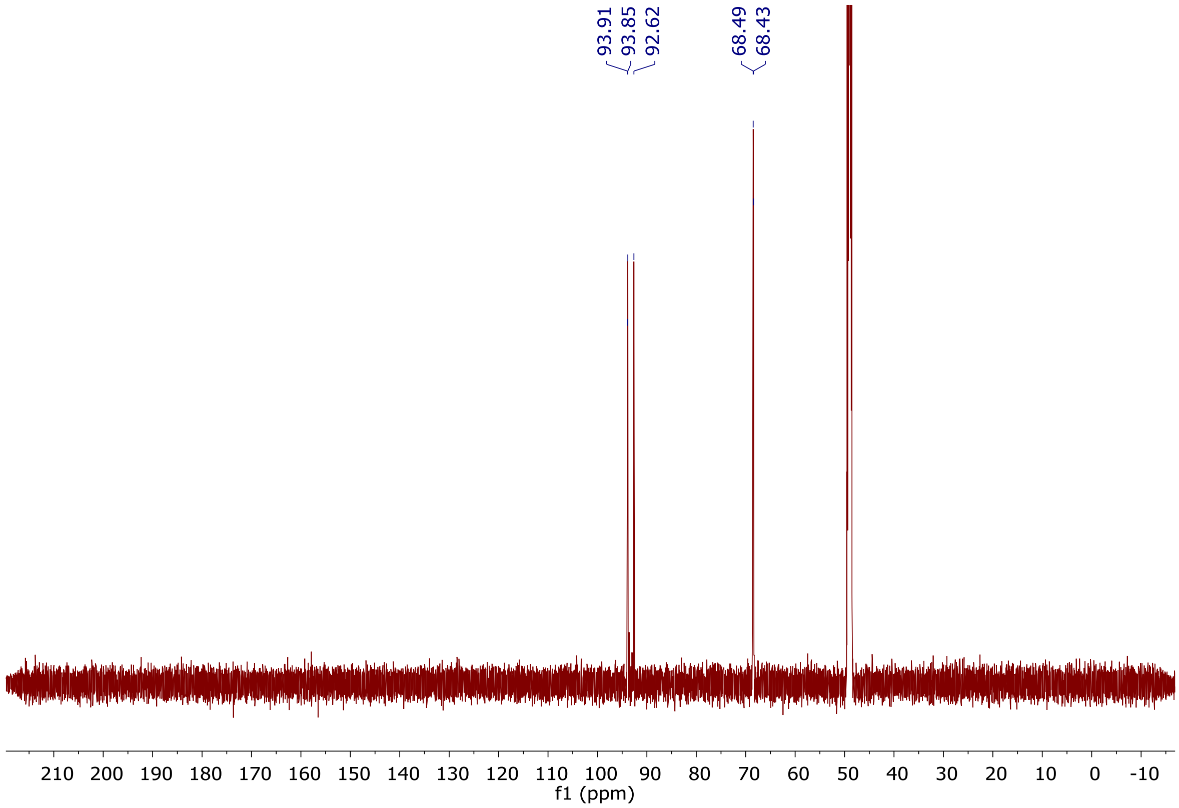

Fig. S17 ^{13}C NMR (151 MHz, 298 K, methanol-d_4) of **3**

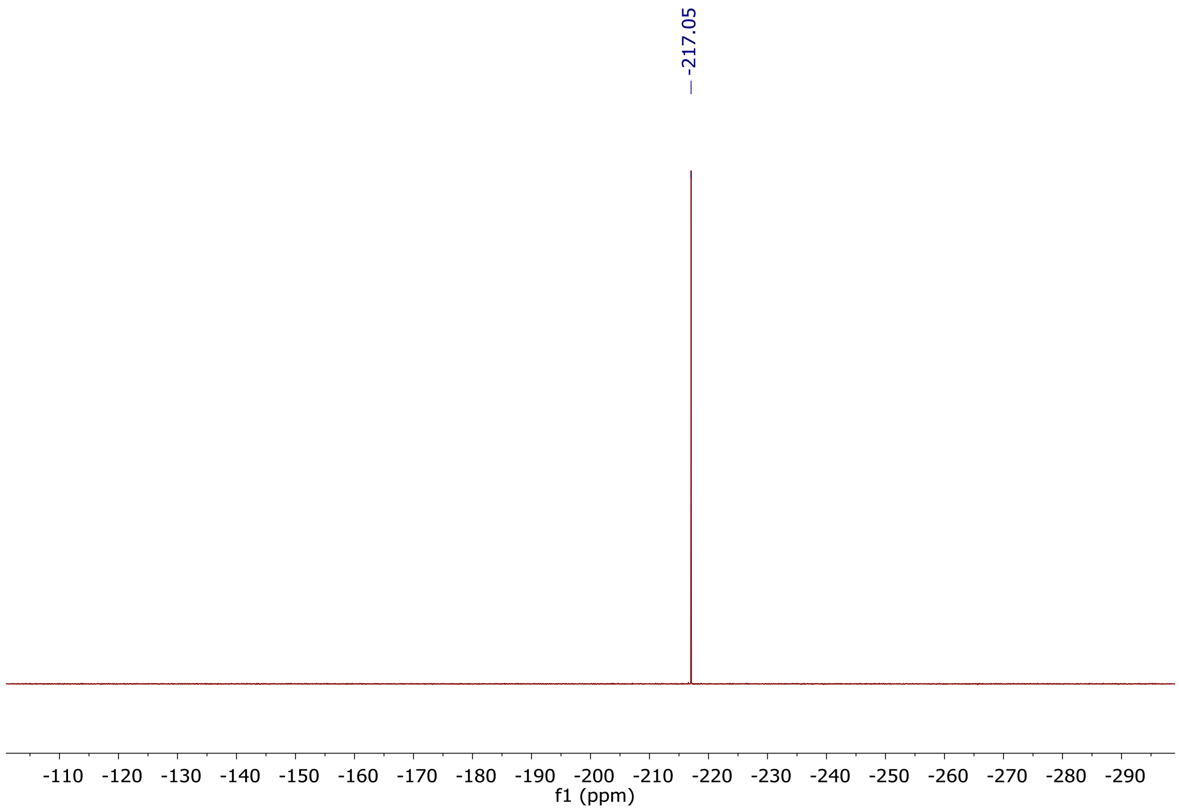

Fig. S18 ^{19}F NMR (376 MHz, 298 K, methanol-d_4) of **3**

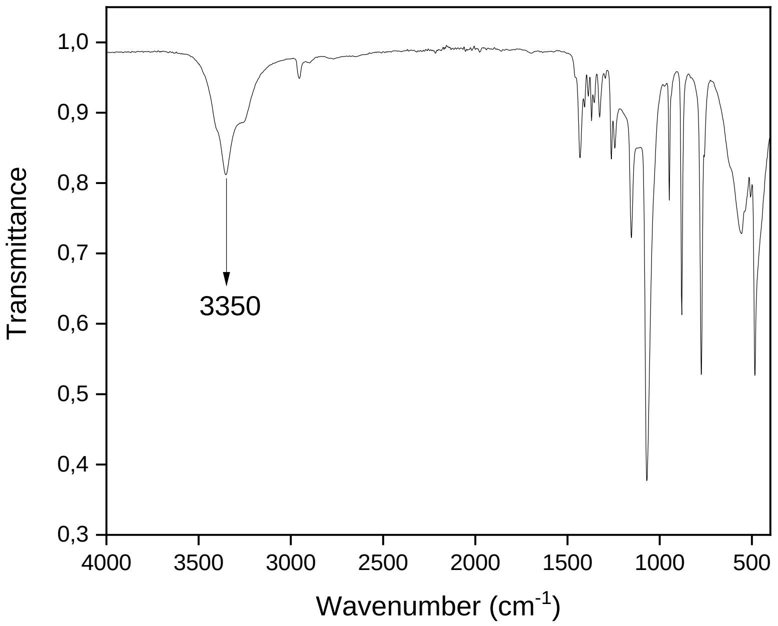

Fig. S19 ATR-IR spectrum of **3** (solid state).

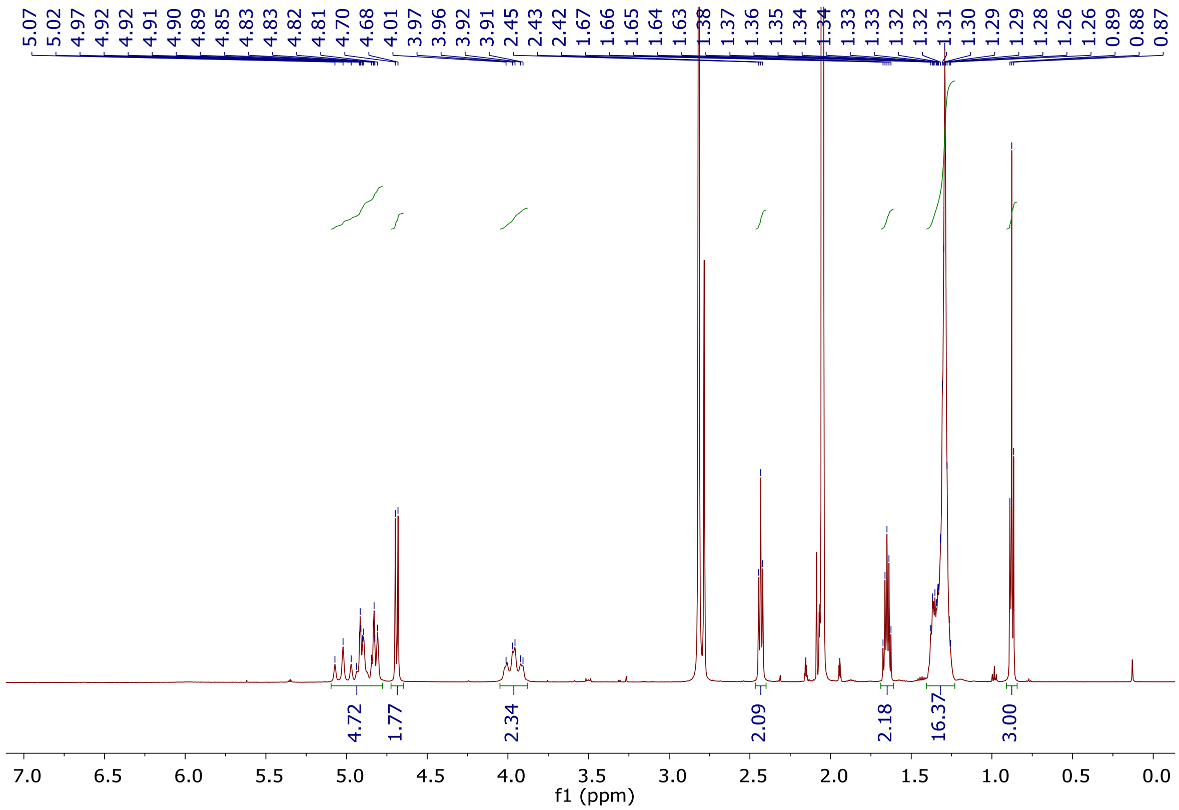

Fig. S20 ^{1}H NMR (600 MHz, 298 K, acetone-d_6) of **4**

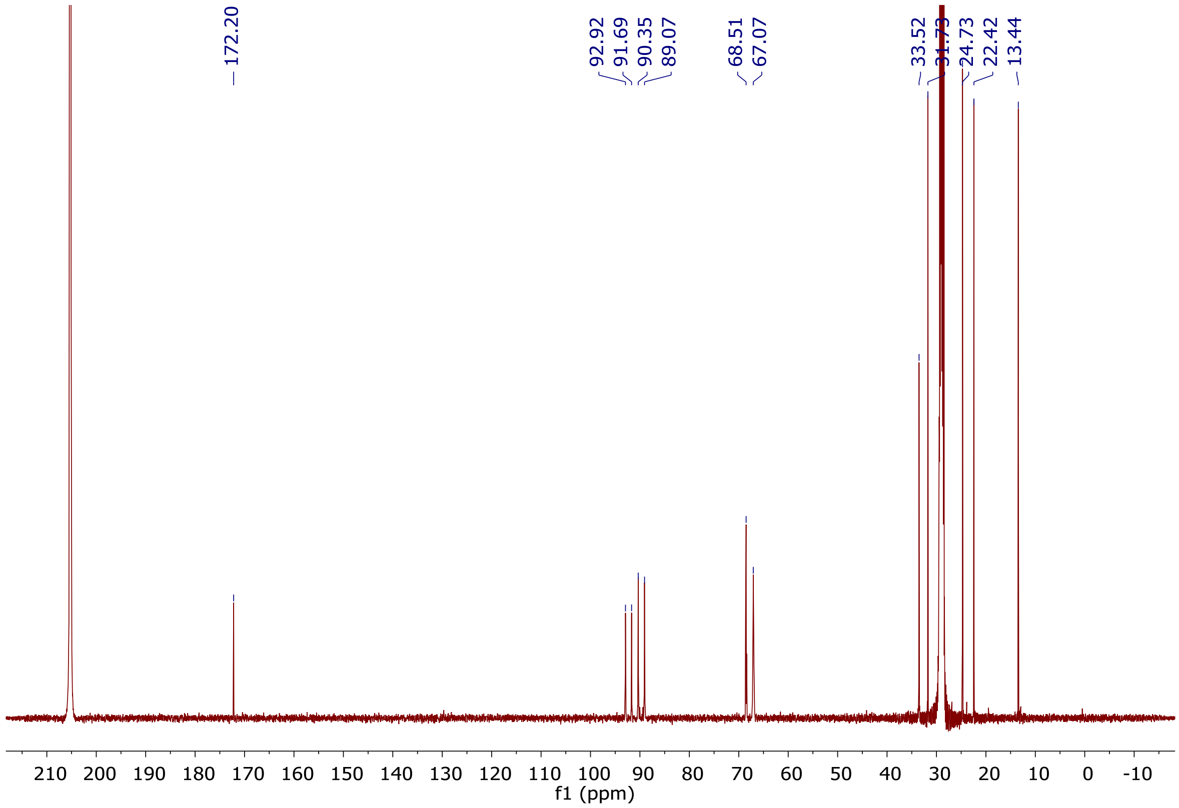

Fig. S21 ^{13}C NMR (151 MHz, 298 K, acetone-d_6) of **4**

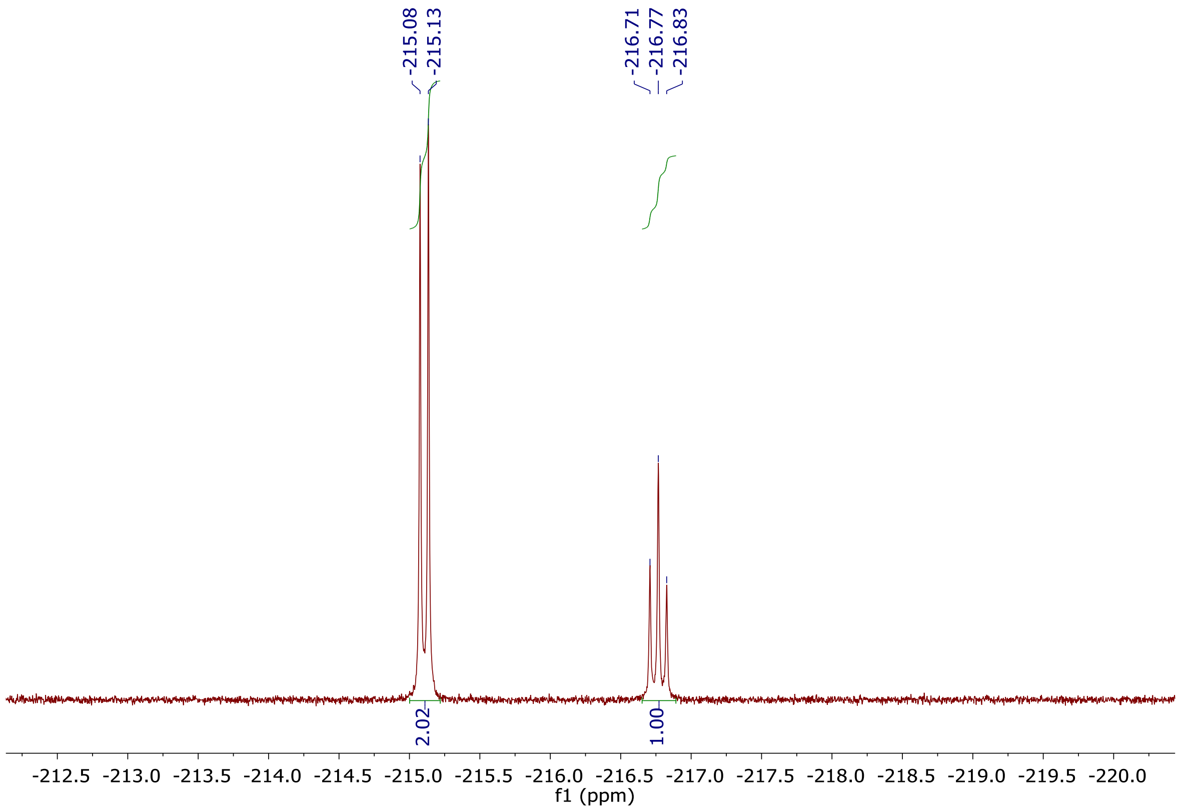

Fig. S22 ^{19}F NMR (376 MHz, 298, K, acetone-d_6) of **4**

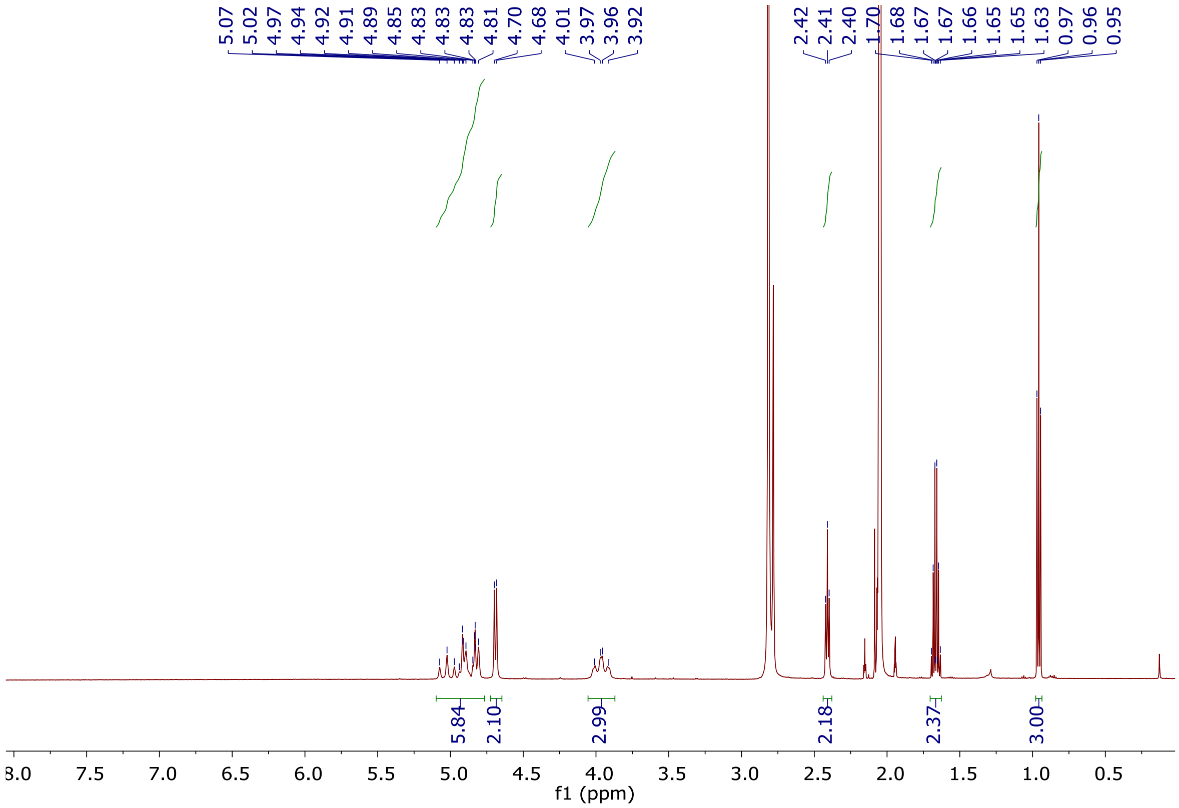

Fig. S23 ^{1}H NMR (600 MHz, 298 K, acetone-d_6) of **5**

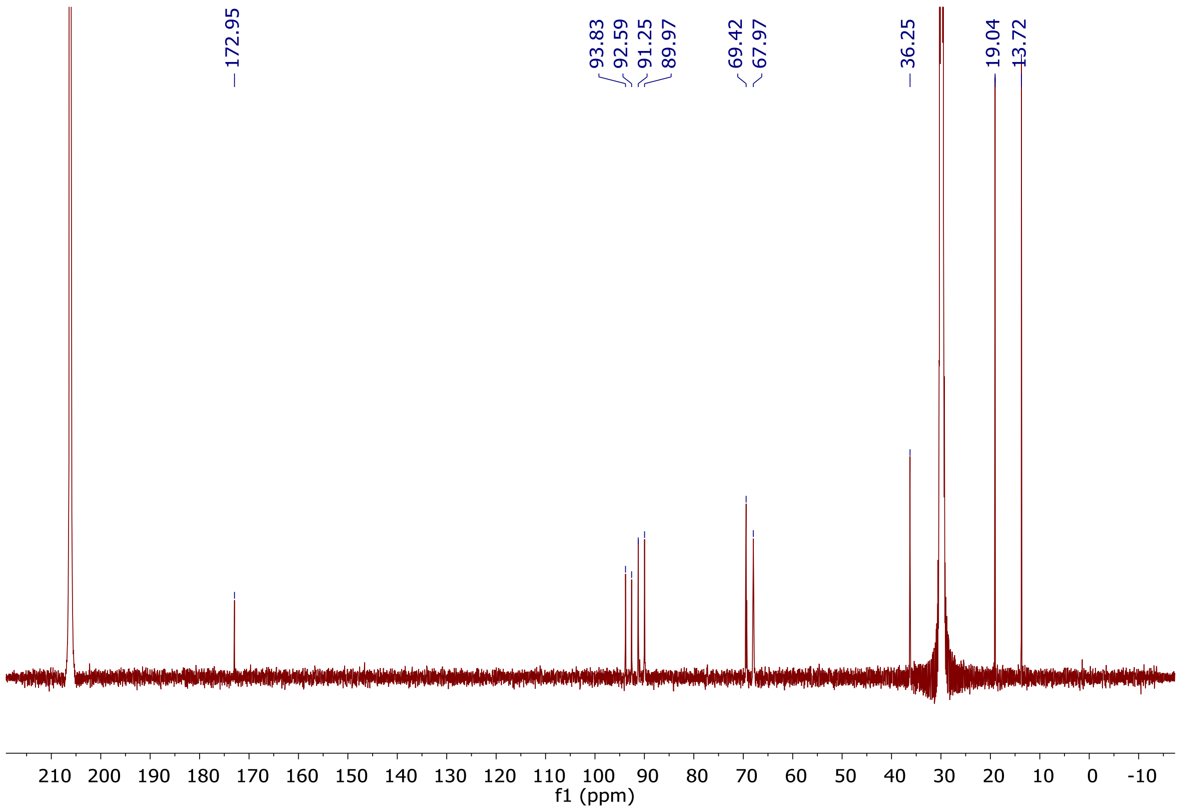

Fig. S24 ^{13}C NMR (151 MHz, 298 K, acetone-d_6) of **5**

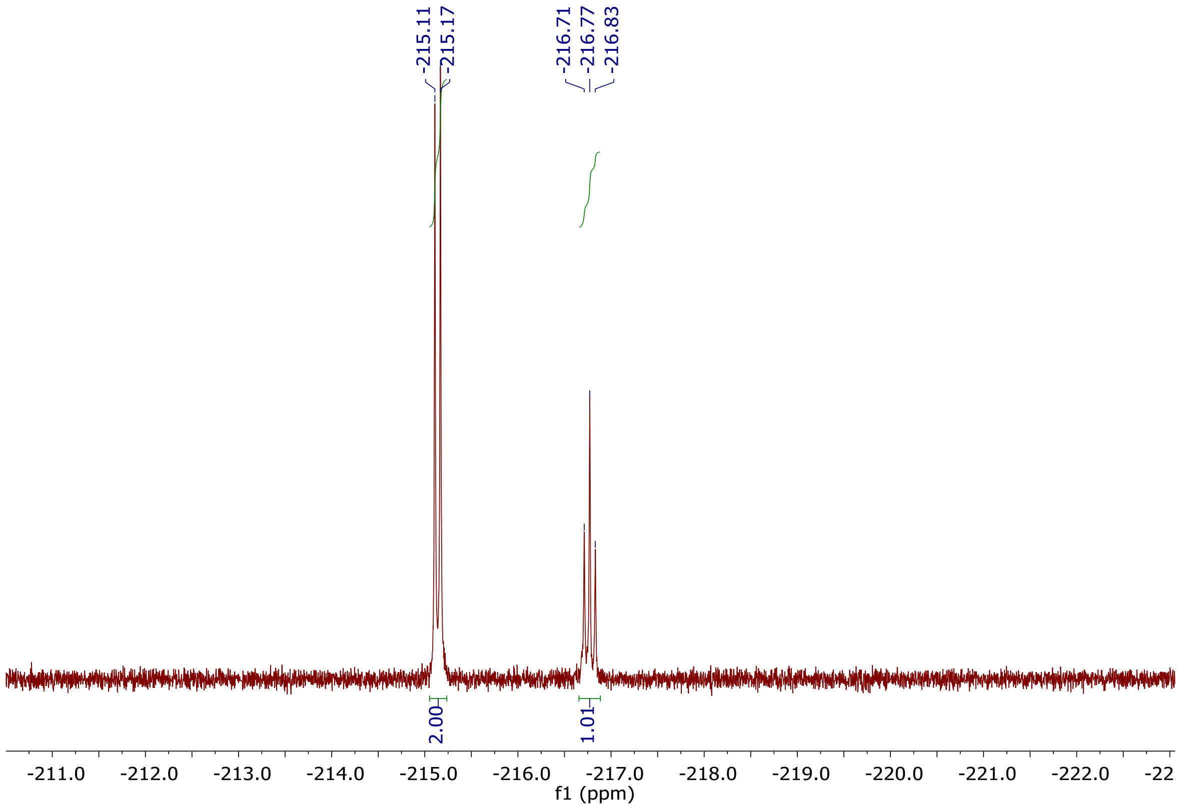

Fig. S25 ^{19}F NMR (376 MHz, 298 K, acetone-d_6) of **5**

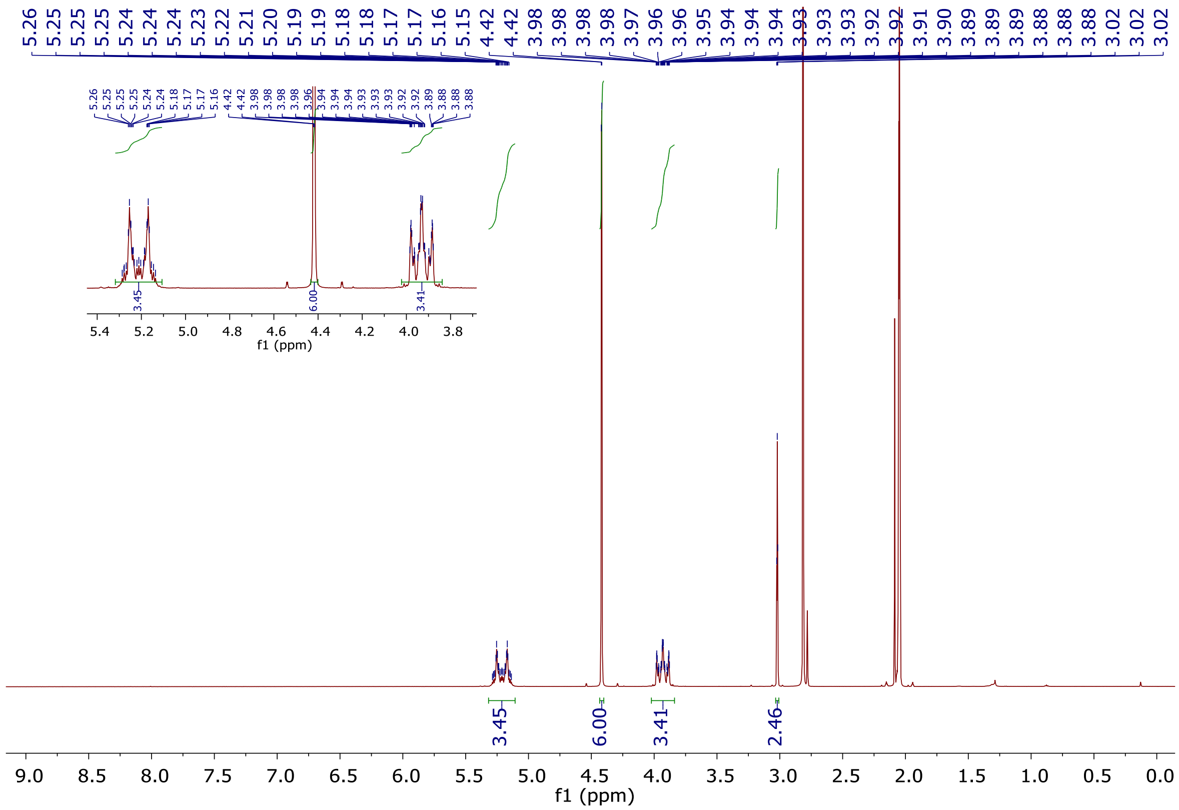

Fig. S26 ^{1}H NMR (600 MHz, 298 K, acetone-d_6) of **6**

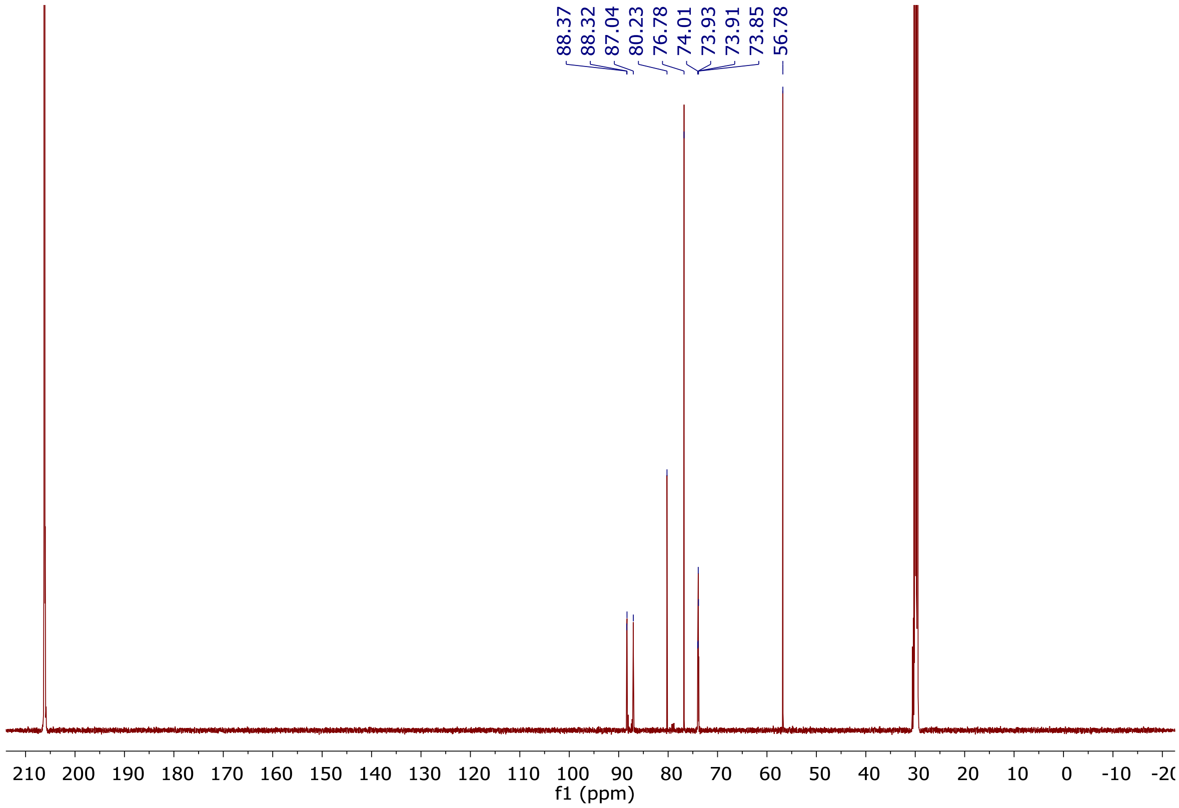

Fig. S27 ^{13}C NMR (151 MHz, 298 K, acetone-d_6) of **6**

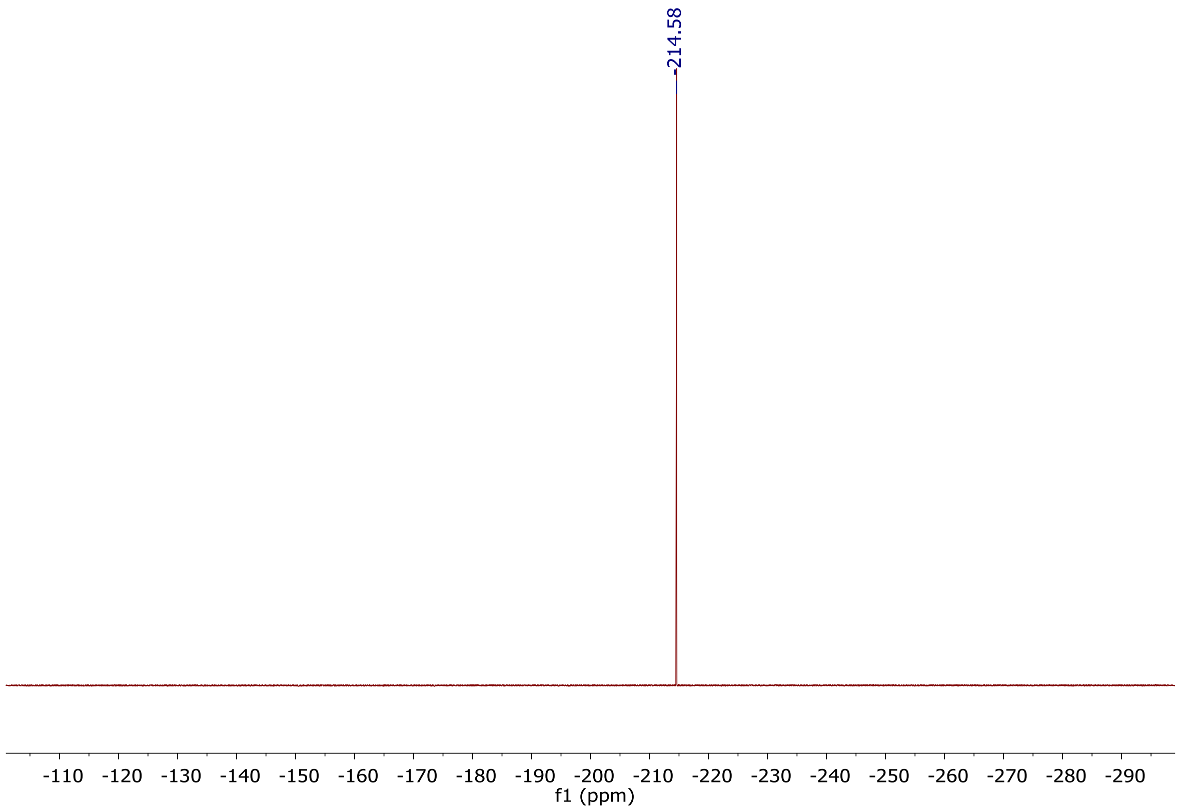

Fig. S28 ^{19}F NMR (376 MHz, 298 K, acetone-d_6) of **6**

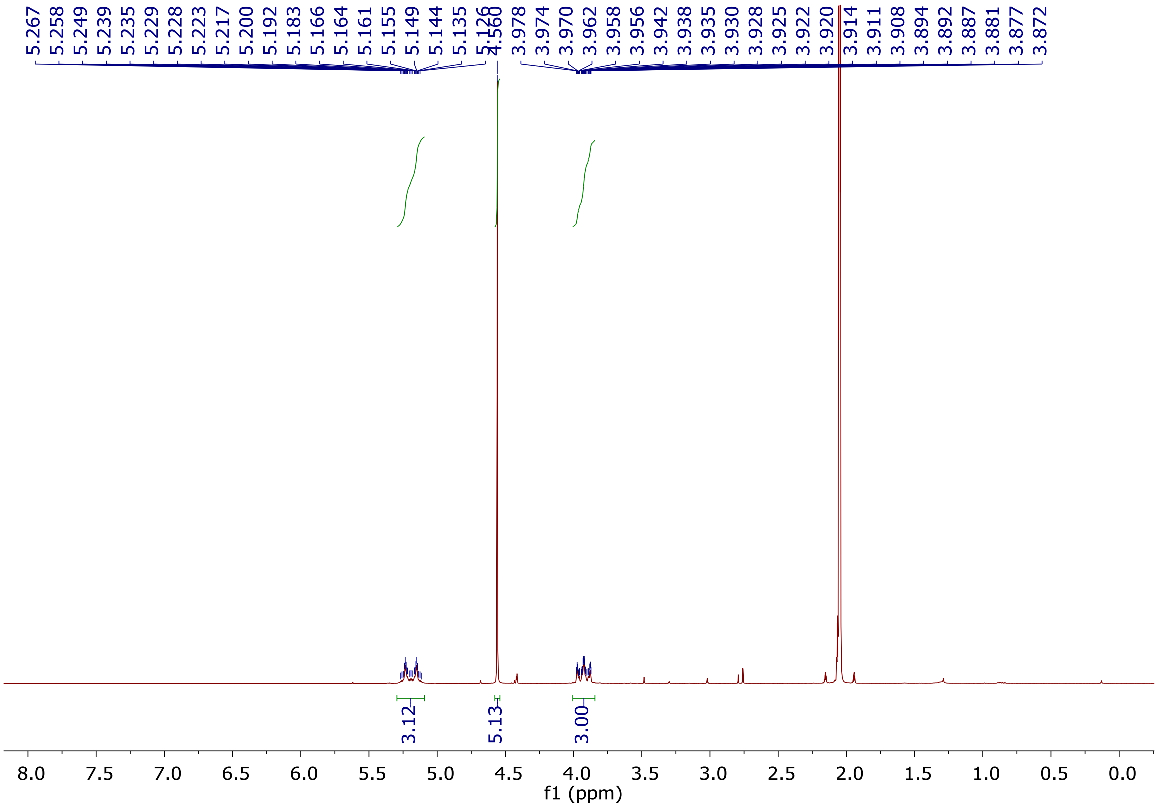

Fig. S29 ^{1}H NMR (600 MHz, 298 K, acetone-d_6) of **7**

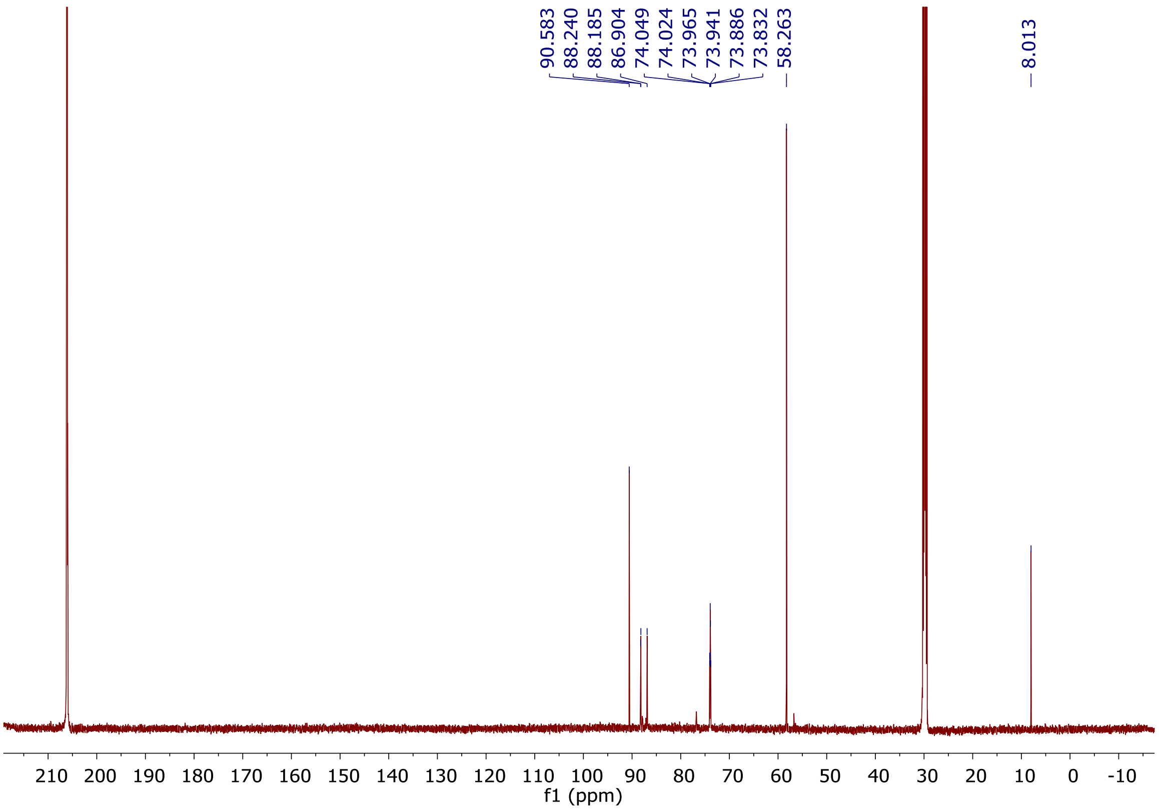

Fig. S30 ^{13}C NMR (151 MHz, 298 K, acetone-d_6) of **7**

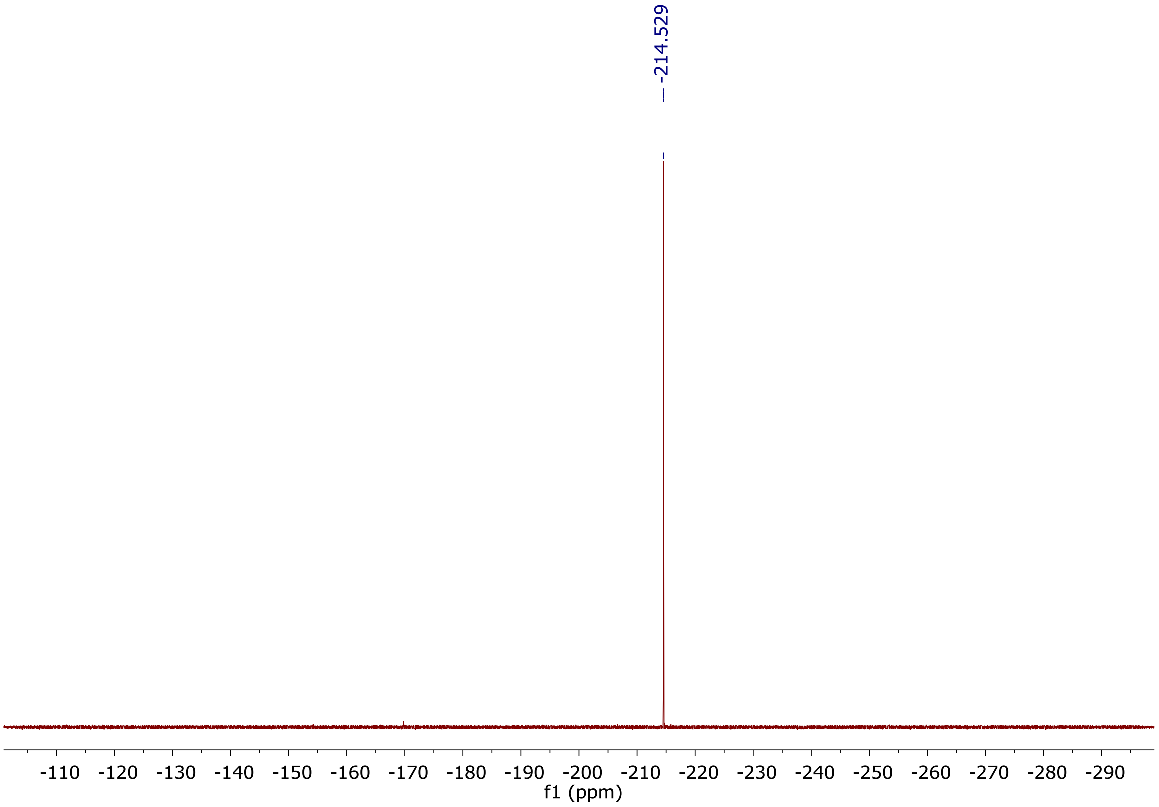

Fig. S31 ^{19}F NMR (376 MHz, 298 K, acetone-d_6) of **7**

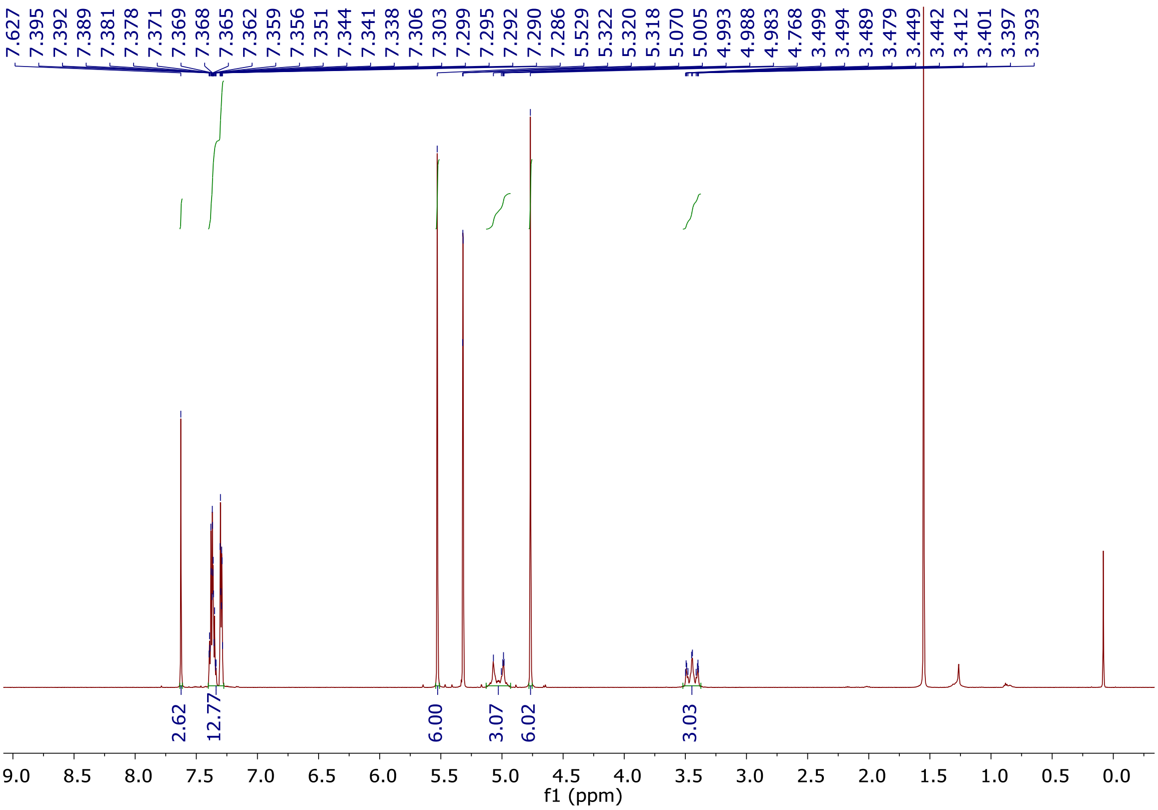

Fig. S32 ^{1}H NMR (600 MHz, 298 K, dichloromethane-d_2) of **8**

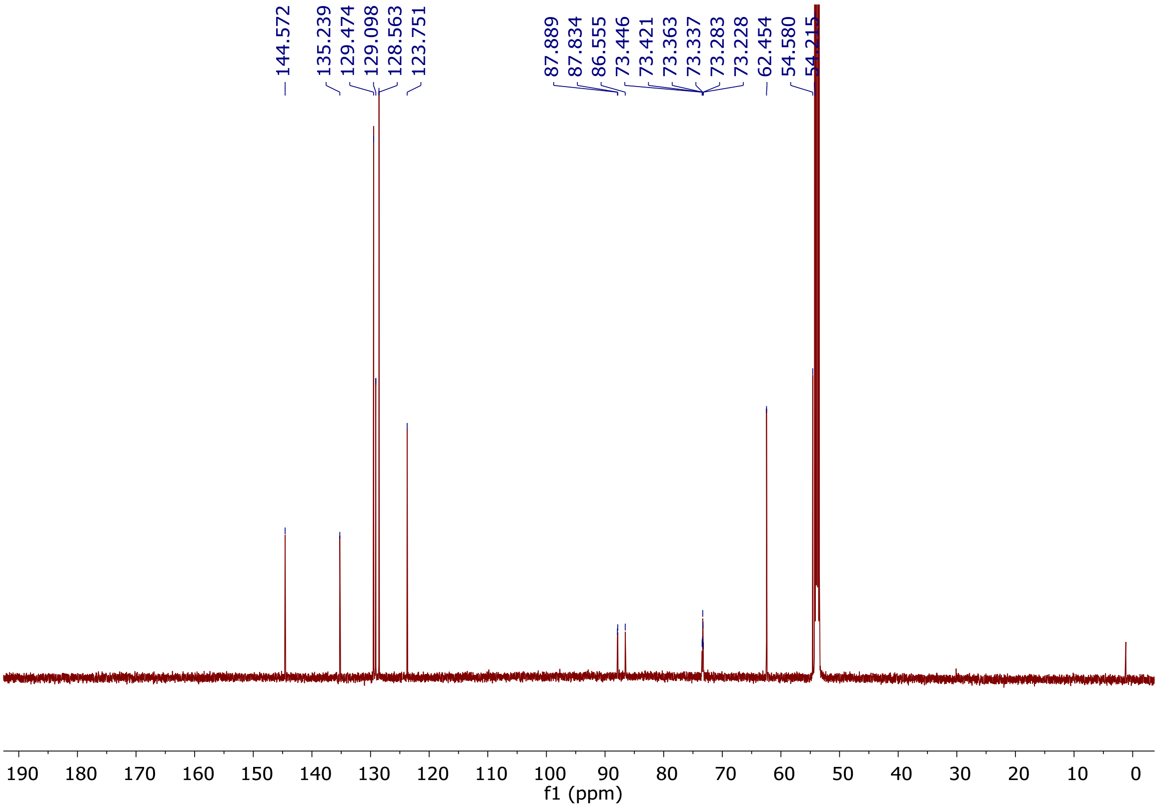

Fig. S33 ^{13}C NMR (151 MHz, 298 K, dichloromethane-d_2) of **8**

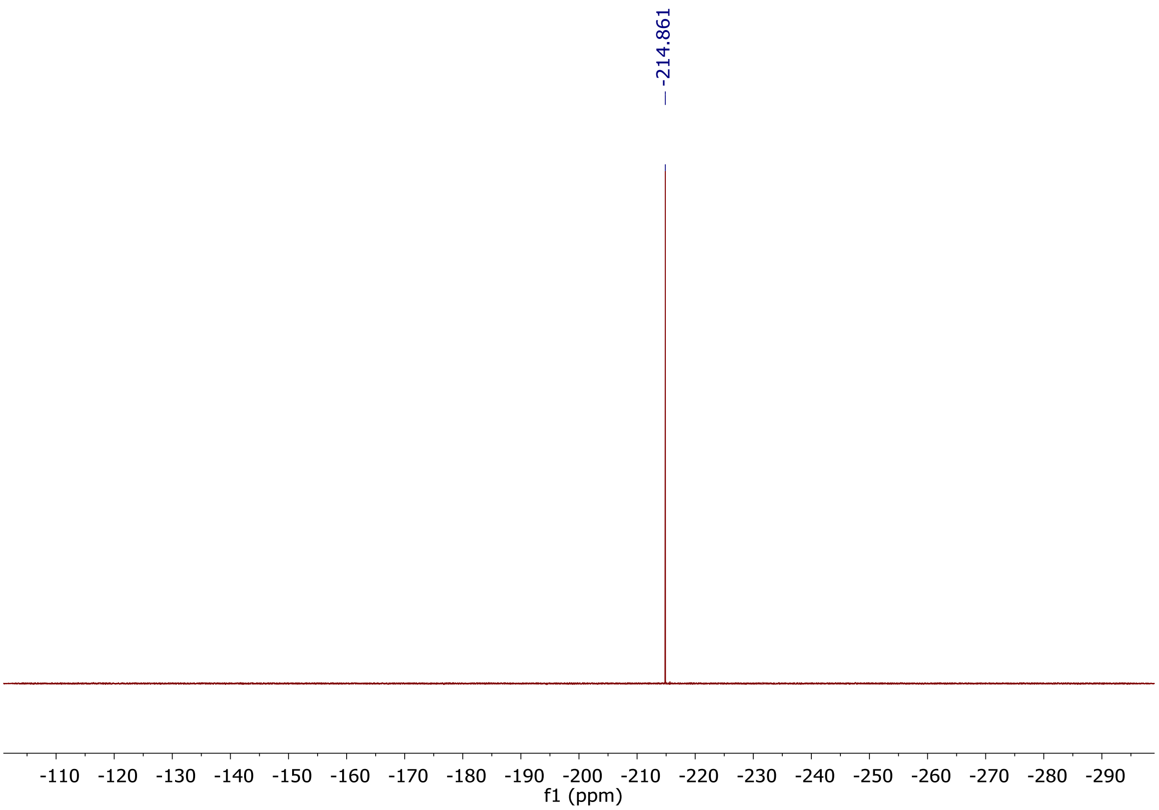

Fig. S34 ^{19}F NMR (376 MHz, 298 K, dichloromethane-d_2) of **8**

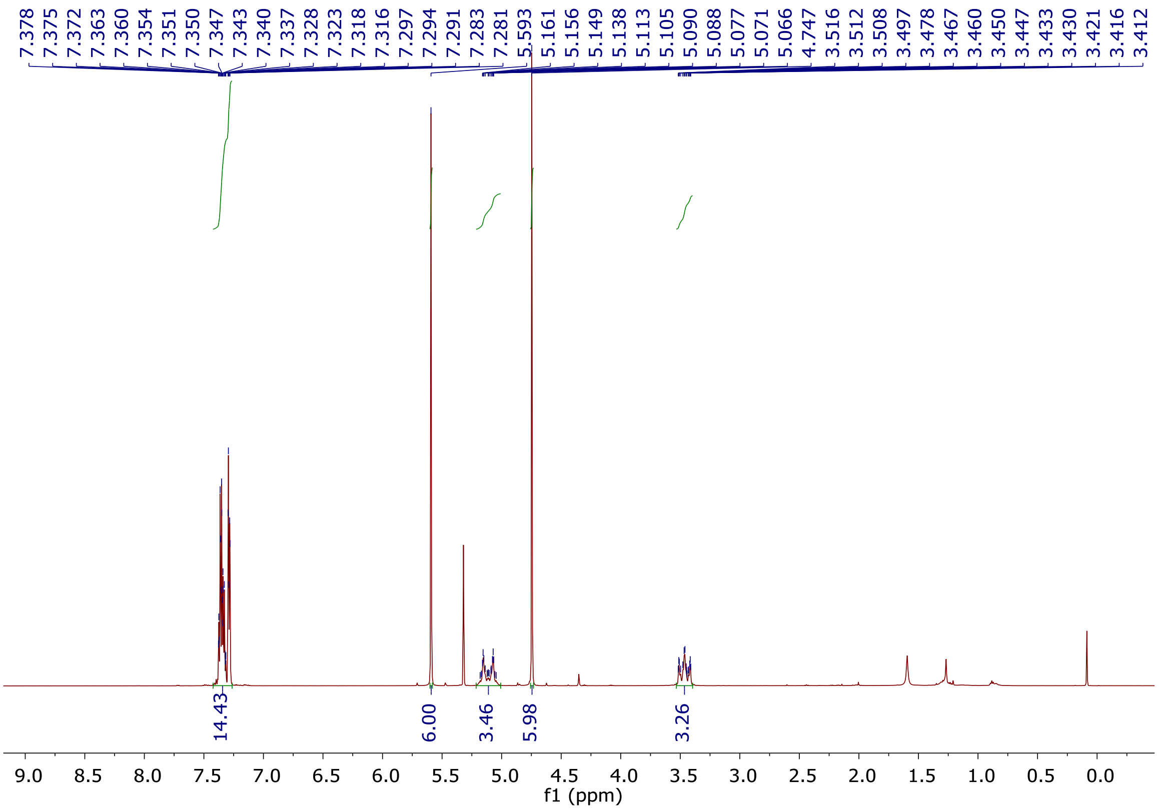

Fig. S35 ^{1}H NMR (600 MHz, 298 K, dichloromethane-d_2) of **9**

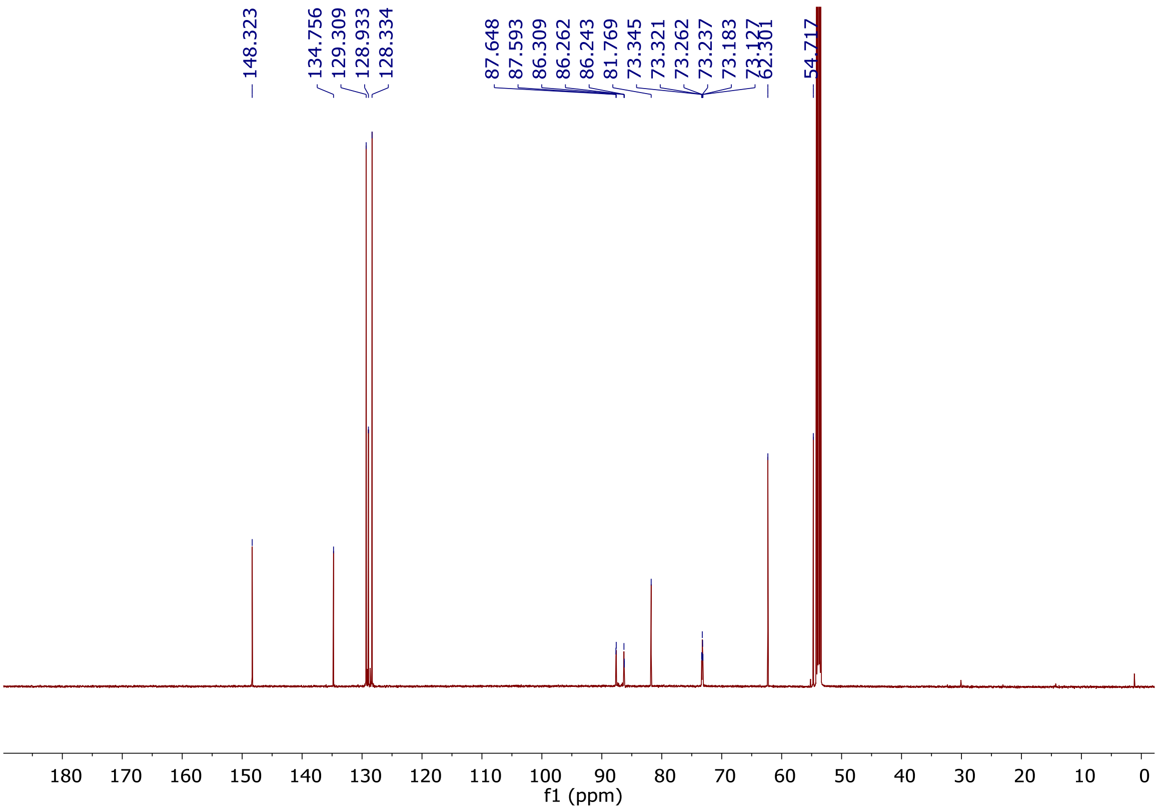

Fig. S36 ^{13}C NMR (151 MHz, 298 K, dichloromethane-d_2) of **9**

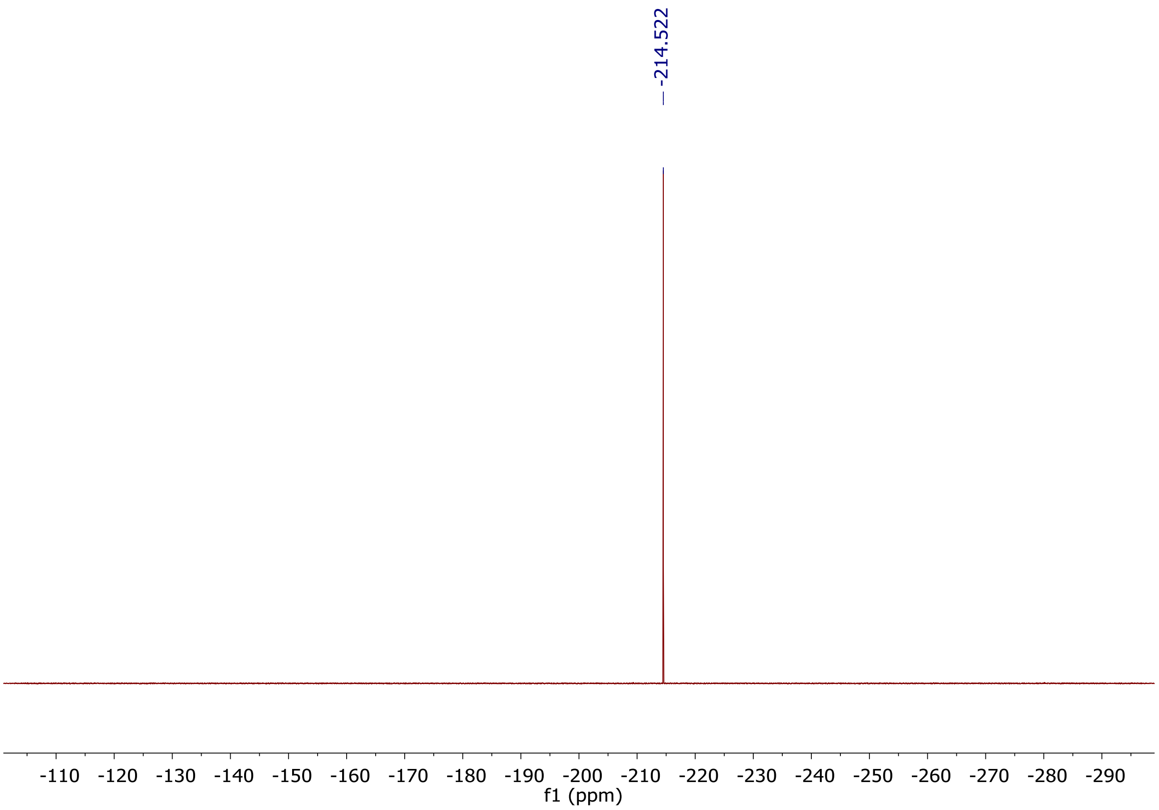

Fig. S37 ^{19}F NMR (376 MHz, 298 K, dichloromethane-d_2) of **9**

8. Crystallographic data

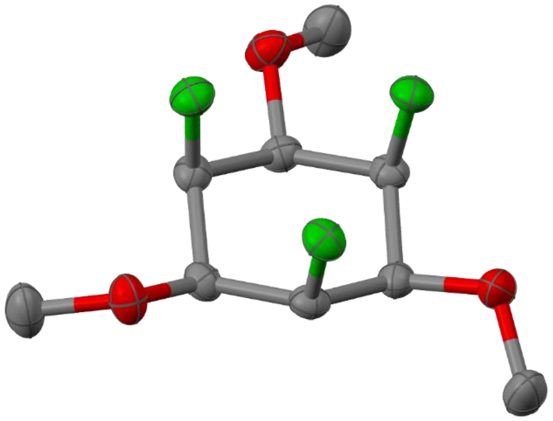

Fig. S38. SCXRD refined structure of **2** (SVH21023)

CCDC number: 2306049

Needle like crystals of **2** suitable for diffraction were obtained by slow evaporation of acetone solution and measured at 150 K.

Table S6 Crystal data and structure refinement for SVH21023[13-15].

Identification code	SVH21023
Empirical formula	$C_9H_{15}F_3O_3$
Formula weight	228.21
Temperature/K	150.00
Crystal system	hexagonal
Space group	$P6_3$
a/Å	11.4469(4)
b/Å	11.4469(4)
c/Å	4.7028(2)
α/°	90
β/°	90
γ/°	120
Volume/Å^3	533.66(4)
Z	2
ρ_{calc}g/cm^3	1.420
μ/mm^{-1}	1.209
F(000)	240.0
Crystal size/mm^3	0.488 × 0.097 × 0.074
Radiation	Cu Kα (λ = 1.54184)
2Θ range for data collection/°	8.92 to 145.718
Index ranges	-14 ≤ h ≤ 13, -13 ≤ k ≤ 14, -5 ≤ l ≤ 3
Reflections collected	3334
Independent reflections	548 [R_{int} = 0.0353, R_{sigma} = 0.0196]

Data/restraints/parameters	548/1/47
Goodness-of-fit on F^2	1.105
Final R indexes [I>=2σ (I)]	R_1 = 0.0270, wR_2 = 0.0697
Final R indexes [all data]	R_1 = 0.0306, wR_2 = 0.0726
Largest diff. peak/hole / e Å^{-3}	0.17/-0.14
Flack parameter	0.38(18)

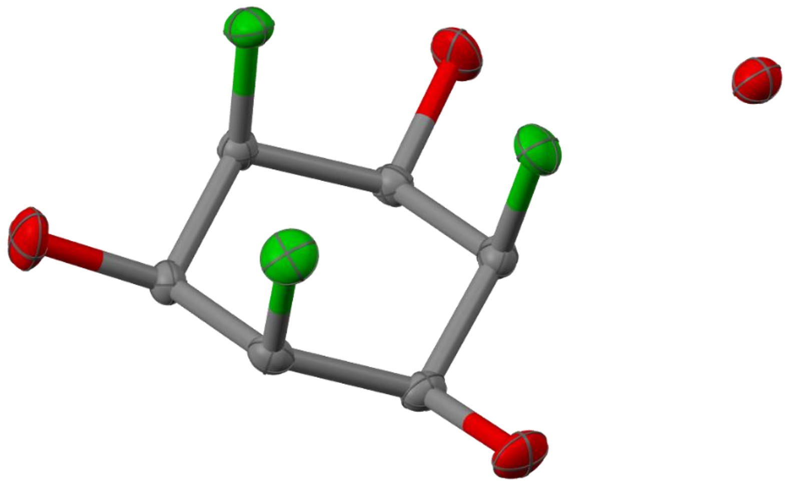

Fig. S39. SCXRD refined structure of **3** (SVH21049)

CCDC number: 2306050

Needle like crystals of **3** suitable for diffraction were obtained by recrystallization in acetone and measured at 150 K.

Table S7. Crystal data and structure refinement for SVH21049.

Identification code	SVH21049
Empirical formula	$C_6H_{11}F_3O_4$
Formula weight	204.15
Temperature/K	149.95
Crystal system	orthorhombic
Space group	Pnma
a/Å	17.1696(5)
b/Å	9.4705(3)
c/Å	4.82980(10)
α/°	90
β/°	90
γ/°	90
Volume/Å^3	785.35(4)
Z	4
ρ_{calc}g/cm^3	1.727
μ/mm^{-1}	1.659
F(000)	424.0
Crystal size/mm^3	0.519 × 0.141 × 0.09

Radiation	Cu Kα (λ = 1.54184)
2Θ range for data collection/°	10.304 to 146.088
Index ranges	-20 ≤ h ≤ 14, -11 ≤ k ≤ 10, -5 ≤ l ≤ 5
Reflections collected	4748
Independent reflections	821 [R_{int} = 0.0396, R_{sigma} = 0.0212]
Data/restraints/parameters	821/0/72
Goodness-of-fit on F^2	1.110
Final R indexes [I>=2σ (I)]	R_1 = 0.0357, wR_2 = 0.0947
Final R indexes [all data]	R_1 = 0.0383, wR_2 = 0.0982
Largest diff. peak/hole / e Å^{-3}	0.36/-0.39

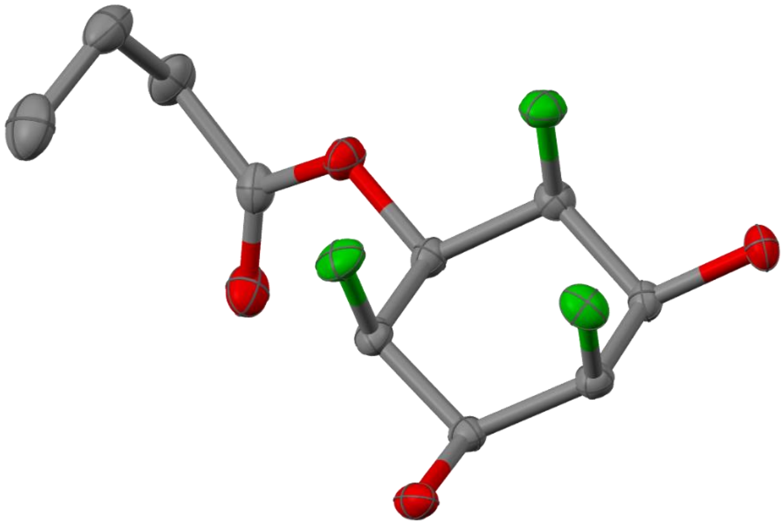

Fig. S40. SCXRD refined structure of **5** (SVH22076)

CCDC number: 2306058

Crystals of **5** suitable for diffraction were obtained by layering hexane over a methanol solution of **5** and measured at 150 K.

Table S8. Crystal data and structure refinement for SVH22076.

Identification code	SVH22076
Empirical formula	$C_{10}H_{15}F_3O_4$
Formula weight	256.22
Temperature/K	150.00(14)
Crystal system	monoclinic
Space group	$P2_1/n$
a/Å	6.47780(10)
b/Å	8.90490(10)
c/Å	19.2107(2)
α/°	90
β/°	98.9640(10)

γ/°	90
Volume/Å^3	1094.62(2)
Z	4
ρ_{calc}g/cm^3	1.555
μ/mm^{-1}	1.322
F(000)	536.0
Crystal size/mm^3	0.38 × 0.19 × 0.13
Radiation	Cu Kα (λ = 1.54184)
2Θ range for data collection/°	9.322 to 146.002
Index ranges	-8 ≤ h ≤ 6, -10 ≤ k ≤ 11, -23 ≤ l ≤ 23
Reflections collected	15359
Independent reflections	2170 [R_{int} = 0.0550, R_{sigma} = 0.0222]
Data/restraints/parameters	2170/0/158
Goodness-of-fit on F^2	1.050
Final R indexes [I>=2σ (I)]	R_1 = 0.0324, wR_2 = 0.0883
Final R indexes [all data]	R_1 = 0.0339, wR_2 = 0.0897
Largest diff. peak/hole / e Å^{-3}	0.34/-0.22

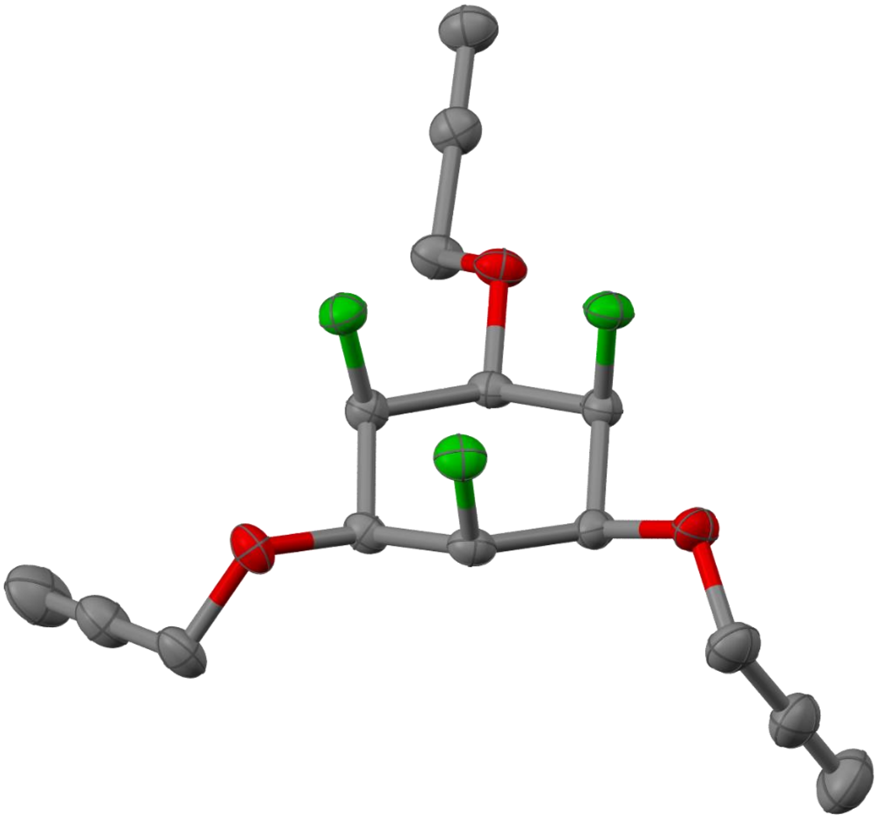

Fig. S41. SCXRD refined structure of **6** (SVH23010_auto)

CCDC number: 2306069

Crystals of **6** suitable for diffraction were obtained by slow evaporation of dichloromethane solution and measured at 150 K.

Table S9. Crystal data and structure refinement for SVH23010_auto.

Identification code	SVH23010_auto
Empirical formula	$C_{15}H_{15}F_3O_3$
Formula weight	300.27
Temperature/K	150.00(14)
Crystal system	monoclinic
Space group	C2/c
a/Å	18.1588(5)
b/Å	9.2165(2)
c/Å	16.7115(5)
α/°	90
β/°	92.384(3)
γ/°	90
Volume/Å^3	2794.43(13)
Z	8
ρ_{calc}g/cm^3	1.427
μ/mm^{-1}	1.078
F(000)	1248.0
Crystal size/mm^3	0.48 × 0.1 × 0.07
Radiation	Cu Kα (λ = 1.54184)
2Θ range for data collection/°	9.75 to 148.99
Index ranges	-22 ≤ h ≤ 21, -7 ≤ k ≤ 11, -19 ≤ l ≤ 20
Reflections collected	14602
Independent reflections	2780 [R_{int} = 0.0687, R_{sigma} = 0.0389]
Data/restraints/parameters	2780/0/191
Goodness-of-fit on F^2	1.318
Final R indexes [I>=2σ (I)]	R_1 = 0.0556, wR_2 = 0.1737
Final R indexes [all data]	R_1 = 0.0677, wR_2 = 0.1785
Largest diff. peak/hole / e Å^{-3}	0.52/-0.28

9. References

1. H. Takata, K. Ono and N. Iwasawa, *Chem. Commun*., 2020, **56**, 5613-5616.
2. R. E. Maleczka, F. Shi, D. Holmes and M. R. Smith, *J. Am. Chem. Soc*., 2003, **125**, 7792-7793.
3. C. Yu, B. A. Piscelli, N. Al-Maharik, D. B. Cordes, A. M. Z. Slawin, R. A. Cormanich and D. OˈHagan, *Chem. Commun*., 2022, **58**, 12855-12858.
4. S. Wang, L. Gong, G. E. Fakhri and J. Wang, *New J. Chem*., 2021, **45**, 16833-16840.
5. P. Wellendorph, J. W. Jaroszewski, S. H. Hansen and H. Franzyk, *Eur. J. Med. Chem*., 2003, **38**, 117-122.
6. Y. Liu, D. Huang, J. Huang and K. Maruoka, *J. Org. Chem*., 2017, **82**, 11865-11871.
7. Gaussian 16, Revision C.01, M. J. Frisch, G. W. Trucks, H. B. Schlegel, G. E. Scuseria, M. A. Robb, J. R. Cheeseman, G. Scalmani, V. Barone, G. A. Petersson, H. Nakatsuji, X. Li, M. Caricato, A. V. Marenich, J. Bloino, B. G. Janesko, R. Gomperts, B. Mennucci, H. P. Hratchian, J. V. Ortiz, A. F. Izmaylov, J. L. Sonnenberg, D. Williams-Young, F. Ding, F. Lipparini, F. Egidi, J. Goings, B. Peng, A. Petrone, T. Henderson, D. Ranasinghe, V. G. Zakrzewski, J. Gao, N. Rega, G. Zheng, W. Liang, M. Hada, M. Ehara, K. Toyota, R. Fukuda, J. Hasegawa, M. Ishida, T. Nakajima, Y. Honda, O. Kitao, H. Nakai, T. Vreven, K. Throssell, J. A. Montgomery, Jr., J. E Peralta, F. Ogliaro, M. J. Bearpark, J. J. Heyd, E. N. Brothers, K. N. Kudin, V. N. Staroverov, T. A. Keith, R. Kobayashi, J. Normand, K. Raghavachari, A. P. Rendell, J. C. Burant, S. S. Iyengar, J. Tomasi, M. Cossi, J. M. Millam, M. Klene, C. Adamo, R. Cammi, J. W. Ochterski, R. L. Martin, K. Morokuma, O. Farkas, J. B. Foresman, and D. J. Fox, Gaussian, Inc., Wallingford CT, 2016.
8. J. Perdew, M. Ernzerhof, K. Burke, *J. Chem. Phys.*, 1996, **105**, 9982.
9. S. Grimme, J. Antony, S. Ehrlich, H. Krieg, *J. Chem. Phys.*, 2010, **132**, 154104.
10. T. J. Poskin, B. A. Piscelli, K. Yoshida, D. B. Cordes, A. M. Z. Slawin, R. A. Cormanich, S. Yamada and D. OˈHagan, *Chem. Commun*., 2022, **58**, 7968-7971.
11. C. Yu, B. A. Piscelli, N. Al-Maharik, D. B. Cordes, A. M. Z. Slawin, R. A. Cormanich and D. OˈHagan, *Chem. Commun*., 2022, **58**, 12855-12858.
12. S. Miertus, E. Scrocco, J. Tomasi, *Chem. Phys*., 1981, **55**, 117-129.
13. D. B. Hibbert and P. Thordarson, *Chem. Commun*., 2016, **52**, 12792-12805
14. V. Dolomanov, L. J. Bourhis, R. J. Gildea, J. A. K. Howard and H. Puschmann, *J. Appl. Cryst*., 2009, **42**, 339-341.
15. G. M. Sheldrick, *Acta Cryst*. 2015, **A71**, 3-8.
16. G. M. Sheldrick, *Acta Cryst*. 2015, **C71**, 3-8.
